# Roles of bacteriophages, plasmids and CRISPR immunity in microbial community dynamics revealed using time-series integrated meta-omics

**DOI:** 10.1038/s41564-020-00794-8

**Published:** 2020-11-02

**Authors:** Susana Martínez Arbas, Shaman Narayanasamy, Malte Herold, Laura A. Lebrun, Michael R. Hoopmann, Sujun Li, Tony J. Lam, Benoît J. Kunath, Nathan D. Hicks, Cindy M. Liu, Lance B. Price, Cedric C. Laczny, John D. Gillece, James M. Schupp, Paul S. Keim, Robert L. Moritz, Karoline Faust, Haixu Tang, Yuzhen Ye, Alexander Skupin, Patrick May, Emilie E. L. Muller, Paul Wilmes

**Affiliations:** 1 Luxembourg Centre for Systems Biomedicine, University of Luxembourg, Esch-sur-Alzette, Luxembourg; 2 Institute for Systems Biology, Seattle, WA USA; 3 School of Informatics, Computing and Engineering, Indiana University, Bloomington, IN USA; 4 TGen North, Flagstaff, AZ USA; 5 The Pathogen and Microbiome Institute, Northern Arizona University, Flagstaff, AZ USA; 6 Laboratory of Molecular Bacteriology, KU Leuven, Leuven, Belgium; 7 Department of Neuroscience, University of California, La Jolla, CA USA; 8 Department of Microbiology, Genomics and the Environment, UMR 7156 UNISTRA-CNRS, Université de Strasbourg, Strasbourg, France; 9 Department of Life Sciences and Medicine, Faculty of Science, Technology and Medicine, University of Luxembourg, Esch-sur-Alzette, Luxembourg; 10 Present Address: Megeno S.A., Esch-sur-Alzette, Luxembourg; 11 Present Address: Harvard T.H. Chan School of Public Health, Harvard University, Boston, MA USA; 12 Present Address: Department of Environmental and Occupational Health, Miken Institute School of Public Health, George Washington University, Washington, DC, USA

**Keywords:** Network topology, Ecosystem ecology, Metagenomics, Microbiome, Bacteriology

## Abstract

Viruses and plasmids (invasive mobile genetic elements (iMGEs)) have important roles in shaping microbial communities, but their dynamic interactions with CRISPR-based immunity remain unresolved. We analysed generation-resolved iMGE–host dynamics spanning one and a half years in a microbial consortium from a biological wastewater treatment plant using integrated meta-omics. We identified 31 bacterial metagenome-assembled genomes encoding complete CRISPR–Cas systems and their corresponding iMGEs. CRISPR-targeted plasmids outnumbered their bacteriophage counterparts by at least fivefold, highlighting the importance of CRISPR-mediated defence against plasmids. Linear modelling of our time-series data revealed that the variation in plasmid abundance over time explained more of the observed community dynamics than phages. Community-scale CRISPR-based plasmid–host and phage–host interaction networks revealed an increase in CRISPR-mediated interactions coinciding with a decrease in the dominant ‘*Candidatus* Microthrix parvicella’ population. Protospacers were enriched in sequences targeting genes involved in the transmission of iMGEs. Understanding the factors shaping the fitness of specific populations is necessary to devise control strategies for undesirable species and to predict or explain community-wide phenotypes.

## Abstract (web summary)

CRISPR-based immunity and microbial community dynamics in a wastewater treatment plant over one and a half years.

## Main

Microbial community dynamics are driven by both abiotic (environmental) and biotic (biological) factors. The latter include mobile genetic elements that move within and/or between genomes^1,2^ and are believed to play an important role in microbial community dynamics^3,4^. More specifically, invasive mobile genetic elements (iMGEs), such as bacteriophages and plasmids, may transfer detrimental or beneficial genetic material to or between hosts^1,2^. Bacteriophages (henceforth referred to as phages) are viruses that specifically infect and replicate within bacteria. Phages are considered to be the most abundant and diverse biological entities with single- or double-stranded DNA or RNA genetic material^5^, and potentially play a role in shaping microbial community structure^6,7^. In contrast, plasmids are generally circular, double-stranded DNA molecules independent of the bacterial chromosome that encode their own origin of replication and are usually found in higher copy numbers^8^. Plasmids represent key components in horizontal gene transfer and are major contributors to the spread of antimicrobial resistance^9^.

Prokaryotic hosts have several defence mechanisms^10^ against iMGE invasion. One notable example is the CRISPR–Cas system, which is an adaptive immune process with mechanisms for acquired immunological memory^1,2^. It consists of genomic regions known as clustered regularly inter-spaced short palindromic repeats (CRISPRs) and a class of proteins referred to as CRISPR-associated (Cas) proteins. CRISPR–Cas systems recognize iMGEs and cleave short subsequences from these iMGEs, called protospacers, which are integrated as spacers within the CRISPR loci of prokaryotic genomes^11–13^. The spacer sequences serve as a genetic memory bank of infection history used to recognize and interfere with future invasions. By exploiting the sequence-based links between spacers and protospacers, specific host populations can be linked to specific iMGEs and to their corresponding invasion events^1,2^.

The present work focuses on a model microbial community in an activated sludge biological wastewater treatment plant (BWWTP), which arguably represents the most widely used biotechnological process on our planet and is an essential component of future integrated energy and matter management strategies^14^. Foaming sludge, which occurs as floating islets on the surface of anoxic treatment tanks and is partially composed of populations of lipid-accumulating microorganisms, is particularly suitable for energy recovery via biodiesel production^15^. These communities also represent good models of microbial ecology because they exhibit medial species richness while at the same time being highly dynamic. Foaming sludge represents a convenient and virtually unlimited source of spatially and temporally resolved samples with complementary detailed physicochemical information^16^. Here, we present a time-resolved, integrated meta-omics analysis aimed at elucidating CRISPR-mediated interactions and dynamics between iMGEs and their hosts. The resolved community and population interactions and dynamics highlight that CRISPR-based immunity within the studied community predominantly targets plasmid sequences.

## Results

### Time-resolved meta-omics of foaming sludge islets

A total of 53 samples of foaming sludge islets from the surface of an anoxic tank were collected from a BWWTP over a period of 578 days. The mean sampling frequency of 8 days (s.d. = 16 days) is equivalent to the doubling time of the dominant population ‘*Candidatus* Microthrix parvicella’ *(M. parvicella*)^17,18^, thereby facilitating the study of population dynamics on a generational timescale. Concomitant DNA, RNA and protein fractions were obtained from each sample^19^, which is critical for coherent downstream systematic measurements and multi-omic data integration^20^. These biomolecular fractions were subjected to deep, high-throughput measurements resulting in time-resolved metagenomics (MG), metatranscriptomics (MT) and metaproteomics (MP) data. A total of 1.5 × 10^9^ MG reads and 1.7 × 10^9^ MT reads underwent sample-specific, large-scale bioinformatics processing, followed by MG and MT de novo co-assembly^21^, yielding a total of 2.1 × 10^7^ contigs (Supplementary Table 1). Additionally, we estimated ~50% average coverage of community members resolved for the individual time points (Supplementary Note 1 and Supplementary Fig. 1). MP datasets yielded a total of 7.6 × 10^6^ mass spectra, whereby a total of 9.6 × 10^7^ redundant peptides were identified per sample using the 3.1 × 10^7^ protein sequences predicted from the co-assembled contigs as the search database (Supplementary Table 2).

Contigs from the co-assembled MG and MT data from each sample were binned, producing a total of 26,524 metagenome-assembled genomes (MAGs) across all samples (Supplementary Table 1), of which 1,364 MAGs were selected for dereplication together with a collection of 85 isolate genomes (Supplementary Note 2). The dereplication process yielded pools of MAGs for which we defined representative MAGs (rMAGs)^22^. These rMAGs underwent taxonomic classification, quality filtering and manual curation to yield a total of 92 rMAGs, which were retained for downstream analyses (Supplementary Table 3). In this work, rMAGs are assumed to represent pools of MAGs resulting from dereplication and are equivalent to populations. Therefore, our population-level analyses are, by default, on the rMAG level unless otherwise specified.

### CRISPR–Cas information over the entire meta-omics dataset

**Fig. 1:**
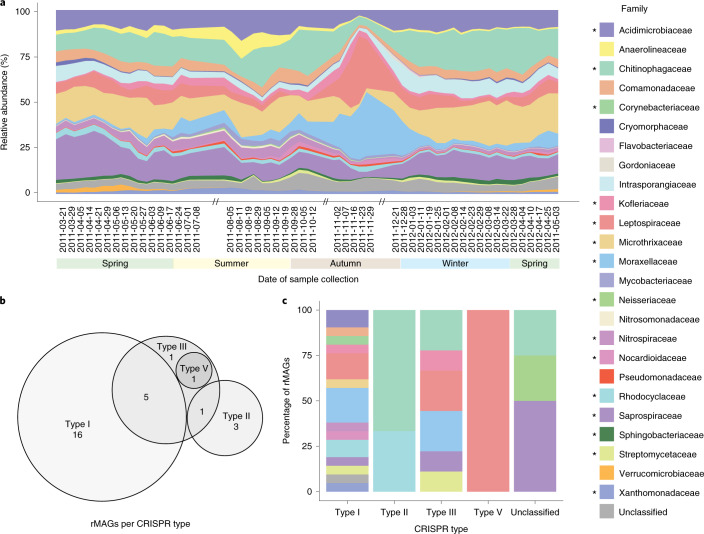
Community dynamics and CRISPR–Cas type distribution. **a**, The relative abundance of rMAGs over time. The labels on the *x* axis indicate the sampling dates and the double slashes (//) on the time axis represent the absence of samples in the sampled system (applicable to all the other figures). **b**, Venn diagram of CRISPR–Cas system types based on the numbers of rMAGs that encode them. Overlaps indicate single rMAGs carrying more than one CRISPR–Cas system. **c**, The distribution of taxonomic affiliations at the family rank per CRISPR–Cas system type. For **a** and **c**, the legend colours marked with asterisks represent families containing CRISPR–Cas systems. Source data

We resolved the CRISPR–Cas systems within rMAGs by extracting their respective *cas* genes and classifying the CRISPR types^23^. This resulted in a final set of 31 (37%) rMAGs that encoded classifiable and complete CRISPR–Cas systems (that is, *cas* genes allowing CRISPR–Cas system classification) and CRISPR loci containing the required information for linking hosts to iMGEs^24^. The most common CRISPR–Cas system within the community was type I, which was found in 21 rMAGs and across several taxonomic families, followed by type III, which was assigned to 9 rMAGs, while type II and V systems were identified in 3 rMAGs and 1 rMAG, respectively. Combinations of different CRISPR types within a single rMAG were also detected. Accordingly, we found that types I and III were present together in five rMAGs, thereby representing the most commonly detected combination^25^ (Fig. 1 and Supplementary Table 4).

**Extended Data Fig. 1:**
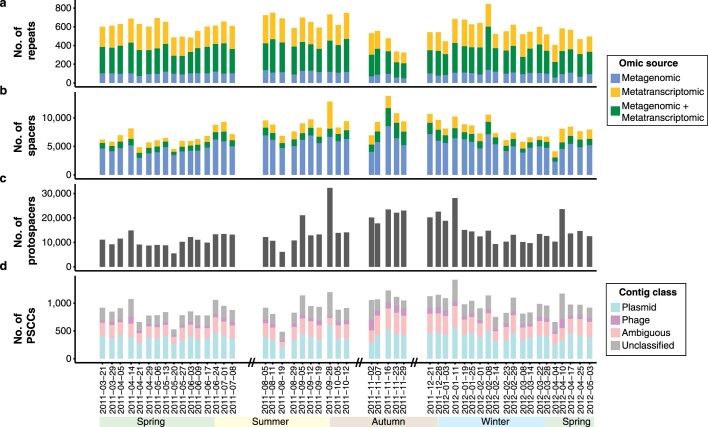
Non-unique CRISPR elements, protospacers, and protospacer-containing contigs (PSCC) over time. Number of predicted **a**, repeats, **b**, spacers, **c**, protospacers, and d, PSCCs per time point. The labels in the x-axis indicate the exact sampling dates, and the double slashes (//) represent absence of samples due to absence of foaming islets. Source data

We used an ensemble of computational methods to extract CRISPR information on the read- and contig- level, which resulted in an extensive set of detected CRISPR repeats and spacers (both collectively referred to as CRISPR elements) per sample. Overall, we retrieved 89,856 repeats and 525,579 spacers over the entire time series. However, they are redundant because the same repeats or spacers may appear at multiple time points (Extended Data Fig. 1). Therefore, we removed redundancy by clustering CRISPR elements, which resulted in 8,469 and 162,985 non-redundant repeats and spacers, respectively. Spacers were more highly represented on the MG level, whereas repeats were more highly represented on the MT level (Supplementary Note 3 and Supplementary Fig. 2). A total of 778 (~9%) non-redundant repeats and 20,002 (~12%) non-redundant spacers could be directly assigned to at least one rMAG, in turn representing 196,159 (~37%) and 29,685 (~33%) redundant spacers and repeats, respectively. To retain the maximum amount of information for downstream analyses, the entire collection of spacers and repeats from the entire pool of MAGs were linked to their corresponding rMAGs (Supplementary Table 4). Although this may result in high numbers of unfiltered spacers associated with certain rMAGs, for example, rMAG-117, which represents 41 MAGs and is associated with 6,574 spacers, this approach allows comprehensive tracking of CRISPR and targeted iMGE dynamics.

### Protospacers in the entire meta-omics dataset

Protospacers may represent either the origin of the spacers or targets for iMGE inhibition/splicing. Spacer information from the CRISPR loci can be used to detect iMGEs through complementary matching to their targeted protospacers^26,27^. Single matches of spacers to targeted iMGEs are considered sufficient for conferring immunity against such iMGEs^28,29^. Thus, spacers were searched against all contigs. Those containing at least one protospacer match, that is, protospacer-containing contigs (hereafter referred to as PSCCs), and lacking repeats to avoid self-matching were considered as putative iMGEs. Accordingly, we detected 750,375 protospacers within 224,651 PSCCs (Extended Data Fig. 1), which highlights the large number of PSCCs that encode multiple protospacers (56%). It is noteworthy that the filtering of PSCCs with repeats (109,504 redundant PSCCs) resulted in the exclusion of potential iMGEs encoding CRISPR loci.

After removing redundancy with the iMGEs (see next section and Supplementary Note 4), a total of 209,199 protospacers were retained within 49,306 non-redundant PSCCs (Supplementary Table 5). Here, there were instances of single spacers targeting multiple protospacers from either different or the same PSCCs. On average, one spacer targeted 21.85 protospacers (median = 7, s.d. = 51.27), while PSCCs tended to contain more than one protospacer (that is, mean = 3.29, median = 2, s.d. = 4.60).

### Plasmids and phages in the entire meta-omics dataset

On the basis of the contigs from all time points, we predicted phage and plasmid sequences. The total number of annotated iMGEs represented 6.97% of all contigs, for which 2.22% contained at least one protospacer (that is, PSCCs). Interestingly, we found that sequences annotated as plasmids outnumbered phages by ~16-fold (Supplementary Note 4 and Supplementary Table 6). At this stage, there was a lack of predicted prophage sequences, which is likely due to limitations of the available phage prediction methods. All the predicted iMGEs were clustered to yield non-redundant representative iMGEs that were traceable over time, which maintained similar proportions to the previously described redundant set; that is, ~16 times more plasmid (707,093) than phages (42,039). Among these, we found 12,232 (1.7%) plasmids and 227 (0.5%) phages with similarities to sequences within the National Center for Biotechnology Information (NCBI) database, which demonstrates the lack of representation of these elements within public databases. A similar trend in proportions was reflected in the iMGEs targeted by spacers. Plasmids (12,412) were targeted five times more frequently than phages (2,351). Since we were interested in iMGEs that are interacting with hosts via CRISPR, we focused on the non-redundant iMGEs that were also PSCCs (henceforth, we collectively refer to these as iMGEs) for downstream analyses. Additionally, the MG and MT co-assembled contigs allowed the detection of iMGEs that were exclusively present on the MT level, for example, RNA phages^30^. Accordingly, a total of 2,890 MT-only contigs assigned as iMGEs were retrieved, from which 2,102 and 387 were classified as plasmid and phage, respectively.

**Extended Data Fig. 2:**
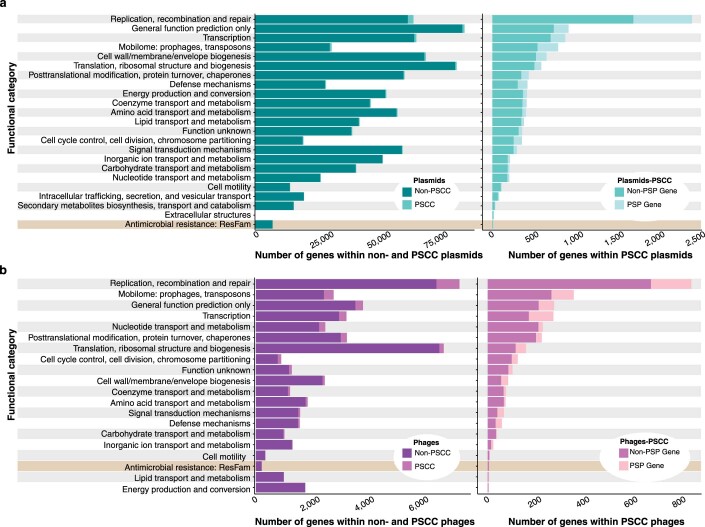
Functional gene categories encoded and targeted within plasmids and phages. **a**, Functional categories encoded by plasmids and **b**, by phages. **a**, **b** Each bar indicates the number of genes found per functional category. The left bar plots show the number of genes of specific functional categories within invasive mobile genetic elements (iMGEs) with and without protospacers (that is PSCCs). For those iMGEs that are PSCCs, the right bar plot highlights the number of genes of specific functional categories for which protospacers occurred within the intragenic regions.

BWWTPs are thought to represent hotspots for the spread of antimicrobial-resistance genes (ARGs)^3,31^. Therefore, we inspected plasmid and phage functions targeted by CRISPR systems^32,33^ and screened those iMGEs for potential ARGs^34^ (Supplementary Note 5, Supplementary Table 7 and Extended Data Fig. 2). We found 1,570 (0.22%) plasmids and 106 (0.25%) phages encoding 38 different ARGs, including tetracycline-resistance genes, which are known to be persistent in BWWTPs^31,35^. Additionally, we found ten plasmid PSCCs. Among these, three encoded ARGs that were being targeted by spacers, specifically aminoglycoside nucleotidyltransferase (ANT3), streptomycin phosphotransferase (APH3′′) and class D beta-lactamases (ClassD) (Supplementary Tables 8 and 9). Apart from these specific cases, iMGEs encoding ARGs were not PSCCs; therefore, they are likely not targeted by CRISPRs.

### Community dynamics

**Extended Data Fig. 3:**
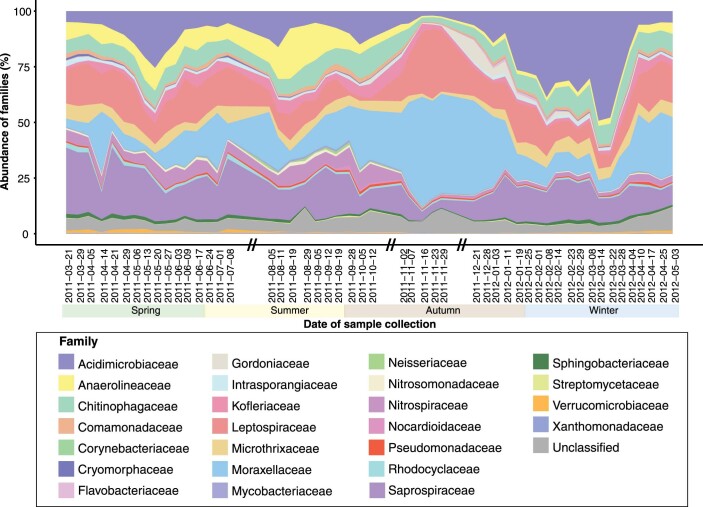
Community activity. Relative expression based on mapping MT data to representative metagenomic assembled genomes (rMAGs) over time. The labels on the x-axis indicate the sampling dates and the double slashes (//) on the time axis represent absence of samples in the sampled system.

The relative abundance of rMAGs and representative iMGEs were used to infer community dynamics over time (Fig. 1, Extended Data Fig. 3 and Supplementary Fig. 3). We grouped rMAGs at the family level due to the large fraction of unclassified taxa. Families such as Microthrixaceae, Moraxellaceae, Leptospiraceae and Acidimicrobiaceae, which are present within sludge communities^15,36^, were prominent members. To further investigate the effects of iMGEs on the community dynamics, we linked iMGEs to their putative host families based on their assignments via binning. This resulted in a total of 79 family-level groups of bacteria, plasmids and phages.

The Microthrixaceae family showed a relative abundance average of 15.5% (median = 15.9%, s.d. = 5.2) with minor fluctuations throughout the time series, except between 2011-11-16 and 2012-01-03, when there was a significant decrease. Moraxellaceae (mean = 6.4%, median = 3.6%, s.d. = 7.5) and Leptospiraceae (mean = 6.9%, median = 5.9%, s.d. = 6.4) showed relatively low abundance over time, but increased with the decline in Microthrixaceae (Fig. 1), thereby representing the shift in the community structure.

**Fig. 2:**
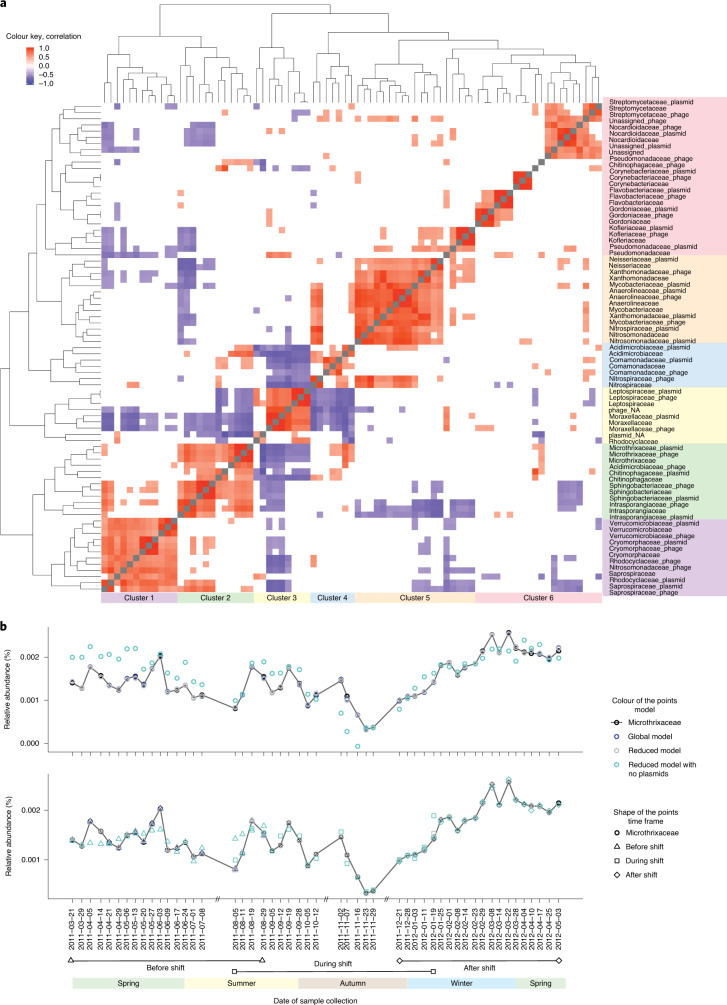
Microbial community dynamics. **a**, The rMAGs were grouped together at the family level. Plasmids and phages were distinctly grouped on the basis of their family-level association (that is, binned together with a rMAG of a given family). The bacterial, plasmid and phage family-level groups were clustered on the basis of the correlation of their group-level abundance dynamics. The groups are displayed on the right of the heatmap. The coloured block on the right and bottom of the heatmap represents the six clusters emerging from the hierarchical clustering, represented by the trees at the top and left of the heatmap. The shown Pearson correlations have a significant level of *P* < 0.001 (that is, threshold). Statistical tests were two-sided and adjusted for multiple comparison. **b**, Upper: models based on the longer-term dynamics. Lower: models based on three shorter-term dynamics. The models are based on the group-level relative abundance values. Longer-term dynamics are represented by all data points from the entire time series. The shorter-term intervals were defined around the shift in community structure, at which the abundance of Microthrixaceae family drastically decreases. Exact sampling dates of the shorter-term intervals are highlighted in the *x* axis. Three models were applied to the longer- and shorter-term time intervals. The relative abundance of the Microthrixaceae family is included for reference. Source data

To further investigate the community dynamics, we defined three overlapping shorter-term intervals according to before, during and after the aforementioned community shift (Fig. 2 and Supplementary Note 6). Subsequently, correlation between the family-level groups, hierarchical clustering and linear modelling using the Microthrixaceae family as the response variable were performed for the entire time series and for shorter-term intervals.

**Extended Data Fig. 4:**
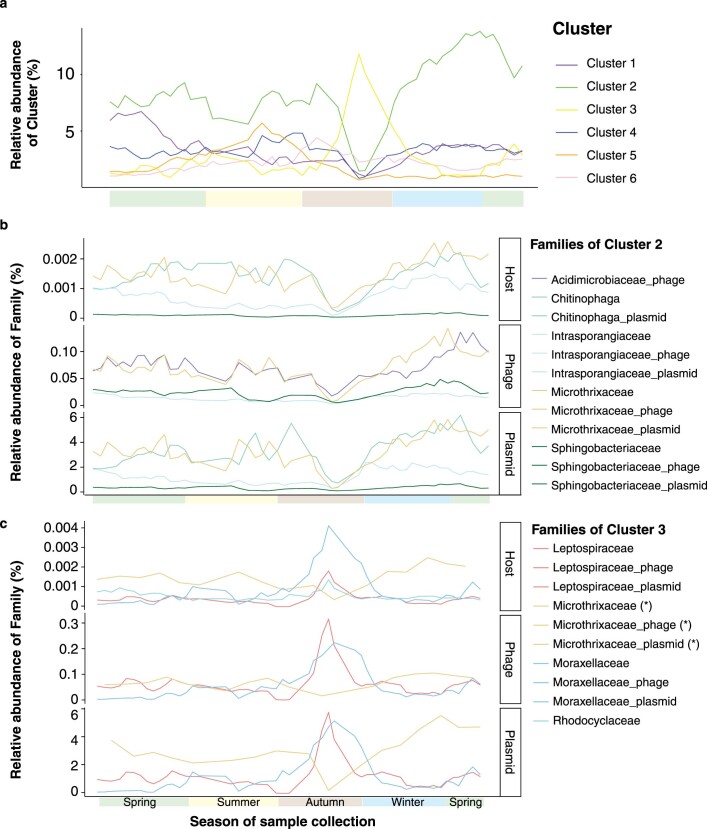
Dynamics of clusters comprised of bacterial-, plasmid- and phage- groups. The rMAGs were grouped together at the family-level. Plasmids and phages were grouped based on their family-level association, that is, binned together with an rMAG of a given family. The bacterial, plasmid and phage groups were clustered based on the correlation of their cumulative group-level abundance dynamics. **a**, Dynamics of all clusters based on cumulative abundance of each cluster members. **b**, Dynamics of the cluster 2 members, including *Microthrixaceae* and its associated plasmids and phages as cluster members. **c**. Dynamics of the cluster 3 members, including *Microthrixaceae* and its associated plasmids and phages as reference (these groups are marked with an asterix). Relative abundance values on the y-axis were derived from MG data. The x-axis represents time, colour coded by seasons as labelled in panel **c**. Please refer to Fig. 1 for the exact sampling dates within the seasons.

The correlation analysis showed 62 pairs of family-level groups that consistently exhibited significant correlations (Supplementary Fig. 4), whereby ten families correlated (*r* ≤ −0.7 or *r* ≥ 0.7, *P* ≤ 0.001) with their own plasmids and phages in the entire time series as well as the shorter-term intervals, for example, Microthrixaceae, Moraxellaceae and Leptospiraceae (Supplementary Table 10). Hierarchical clustering of correlation values from the entire time series yielded a total of six clusters, whereby most bacteria, plasmids and phages assigned to the same families clustered together, which demonstrates that there is predictable variation of these family-level groups. Further inspection of the dominant families showed Microthrixaceae clustering separately from Leptospiraceae and Moraxellaceae. The latter two clustered together and exhibited significant negative correlation with Microthrixaceae (*r* = −0.63, *P* = 8.3 × 10^−7^, and *r* = −0.52, *P* = 9.9 × 10^−5^, respectively), which further supports their observed acyclical behaviour relative to Microthrixaceae (Extended Data Fig. 4 and Supplementary Fig. 5).

**Extended Data Fig. 5:**
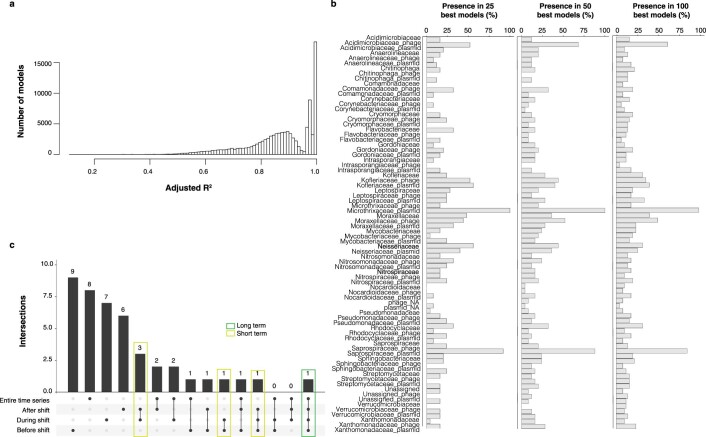
Model fitness and family enrichment within the best models predicting *Microthrixaceae* family abundance. **a**, Distribution of the adjusted R^2^ values of 100,000 model realizations. **b**, Enrichment of the family-level bacterial, plasmid and phage groups in the best 25, 50 and 100 models of the entire time-series. **c**, The upset plot represents the number of family-level bacterial, plasmid and phage groups (that is features) within the best model of different time intervals, that is the entire time-series and three time-windows (horizontal bars). The number of intersections between features in the best models in different long- and short-time intervals (vertical bars). The coloured boxes represent the intersections representing short- and long- term time dynamics, respectively.

**Extended Data Fig. 6:**
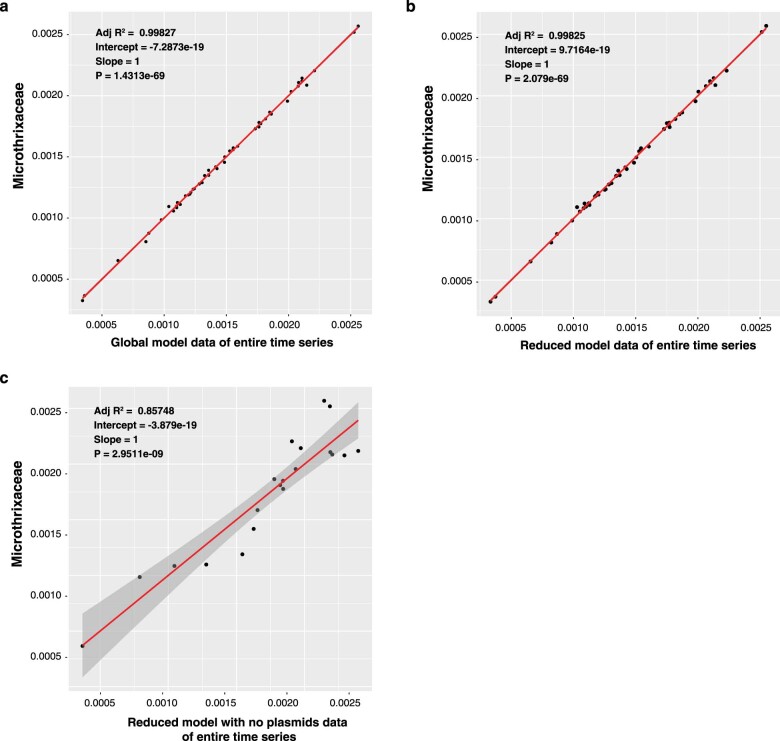
Linear models predicting *Microthrixaceae* family abundance within the entire time-series. Model data fitted to the raw data of the entire time-series (n=51 *in situ* samples), specifically **a**, the best or global model, **b**, the reduced model, which lacks the non-significant families of the global model, and **c**, the reduced model without *Microthrixaceae*-plasmids. Gray bands represent the +/- standard error measurement of the regression line. Statistical tests were two-sided and adjusted for multiple comparisons.

In addition, a selection of the best linear models showed an enrichment of Microthrixaceae plasmids, Acidimicrobiaceae phages and Saprospiraceae plasmids and, in agreement with the enrichment analysis, the best model (adjusted *R*^2^ = 0.9983) showed iMGEs from Microthrixaceae, Saprospiraceae and Moraxellaceae families exhibiting significant contributions (Extended Data Fig. 5). Thus, the longitudinal abundance data for Microthrixaceae exhibited good agreement with the models (Fig. 2). Overall, the linear modelling analysis showed the appearance of Microthrixaceae plasmids as the only common significant predictor in all the models (entire time series and shorter-term intervals). This group was then removed from those models to assess its relative importance, and this resulted in a significant reduction of predictive power (Extended Data Fig. 6, Supplementary Tables 11 and 12, Supplementary Note 7 and Supplementary Fig. 6). Consequently, its plasmids had a stronger effect on the prediction of Microthrixaceae abundance compared to its phages, which indicates a higher relative importance of plasmids in governing Microthrixaceae dynamics.

### CRISPR–Cas mediated iMGE–host interactions

To describe CRISPR-mediated interactions between iMGEs and their hosts, we retained 4,985 spacers that were encoded by at least one rMAG (host), co-occurred with its assigned rMAG in at least one time point and targeted at least one iMGE at any given time point. We subsequently searched for iMGEs and corresponding spacers newly appearing during the time series (that is, spacer integration events), and observed that 2,377 spacers were detected either after or at the same time point as their corresponding targeted iMGEs. The mean spacer integration time (that is, the lag time between the detection of an iMGE and its corresponding spacer) was 9.5 weeks (median = 8, s.d. = 8.5). Spacers that disappeared after the detection of their linked iMGEs were considered to be lost. We observed 1,616 spacers that were lost, with 7 weeks as the average time for such deletions (median = 5.5, s.d. = 7.5). Interestingly, the average time for spacer integration and deletion was lower for phages compared to plasmids (Supplementary Table 13). Furthermore, there was a shift from spacer gain to loss on 2011-11-29, suggesting that the majority of integration events occurred during the summer to autumn transition, while the majority of deletion events occurred in late autumn, which corresponds to the shift in community structure occurring in autumn to winter (Supplementary Fig. 7).

**Fig. 3:**
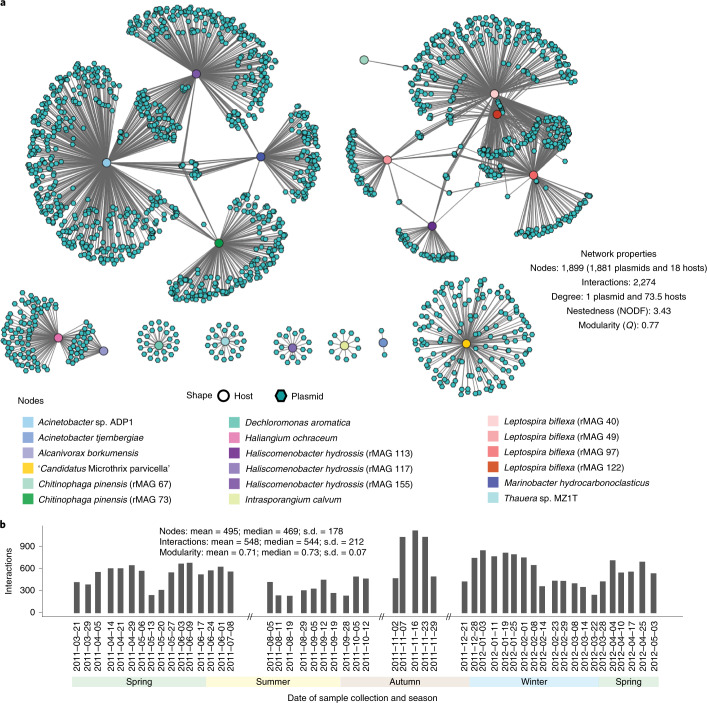
Networks of plasmid–host interactions. **a**, A bipartite network representing global CRISPR-based interactions from the entire time series involving bacterial hosts (multicoloured circular nodes) and their associated plasmids (turquoise hexagonal nodes). The edges represent at least one spacer at one time point from the host targeting the corresponding plasmid. **b**, Number of plasmid–host CRISPR-based interactions. Each bar represents the total number of interactions in a specific time point (*n* = 1), for each of the 51 time points in the time series. The summary statistics within the panel represents the number of CRISPR-based interactions over the entire time series (*n* = 51 in situ samples). Source data

**Extended Data Fig. 7:**
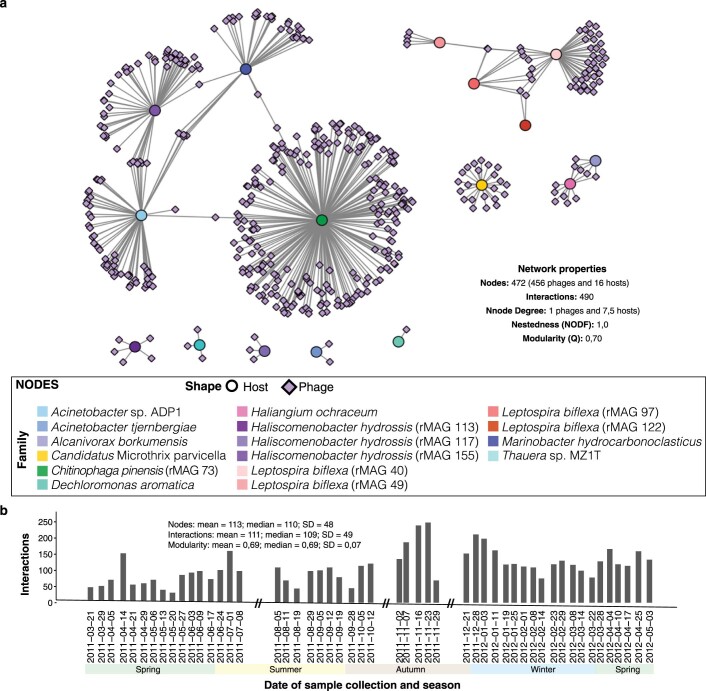
Networks of phage-host interactions. **a**, Bipartite network representing global CRISPR-based interactions from the entire time-series between bacterial hosts (multicolored circular nodes) and their associated phages (purple diamond nodes). The edges represent at least one spacer from the host targeting the corresponding phage throughout the entire time-series. **b**, Number of phage-host CRISPR-based interactions. Each bar represents the total number of interactions in a specific timepoint (n=1), for each of the 51 timepoints in the time-series. The labels on the x-axis indicate the sampling dates and the double slashes (//) on the time axis represent absence of samples in the sampled system. The summary statistics within the panel represents the number of CRISPR-based interactions in the entire time-series (n=51 *in situ* samples). Source data

We then separated the CRISPR-mediated interactions into a plasmid–host network comprising 18 hosts and 1,881 plasmids, with 2,274 interactions (Fig. 3), and a phage–host network comprising 16 hosts and 472 phages, with 490 interactions (Extended Data Fig. 7). We also defined an occurring interaction within a given time point if a host and its interacting iMGE were detected in either MG or MT data, which resulted in time-resolved network topology variations (Supplementary Table 14 and Supplementary Note 8). We included orphan iMGEs and hosts for which their associated counterparts were not detected within the same time point to visualize the dynamics (Supplementary Videos 1 and 2).

The time-resolved plasmid–host interaction networks had an average modularity of *Q* = 0.71 (median = 0.73, s.d. = 0.07), with two main modules of interactions: a group containing a core set of rMAGs classified as *Leptospira biflexa* and a group containing rMAGs from different species, that is, *Marinobacter hydrocarbonoclasticus*, *Acinetobacter* sp. ADP21, *Chitinophaga pinensis* and *Haliscomenobacter hydrossis*. *M. parvicella* was represented by rMAG-165. In contrast, the phage–host interaction networks had an average modularity of *Q* = 0.69 (median = 0.69, s.d. = 0.07) and smaller interacting groups. However, the overall dynamics of both networks were similar, with the number of interactions increasing during November 2011, which co-occurred with the drop in *M. parvicella* (Microthrixaceae) and the increase in other populations, such as *L*. *biflexa* or *H*. *hydrossis*. Based on these networks, we performed a one mode projection to resolve direct interactions between rMAGs with common iMGEs. For this, we observed a higher range of interactions between rMAGs from the plasmid–host network, which suggests that there is a wide spread of plasmids across different families in contrast to the more restricted infection range of phages (Supplementary Fig. 8 and Supplementary Table 15).

### Population-level iMGE–host dynamics

**Fig. 4:**
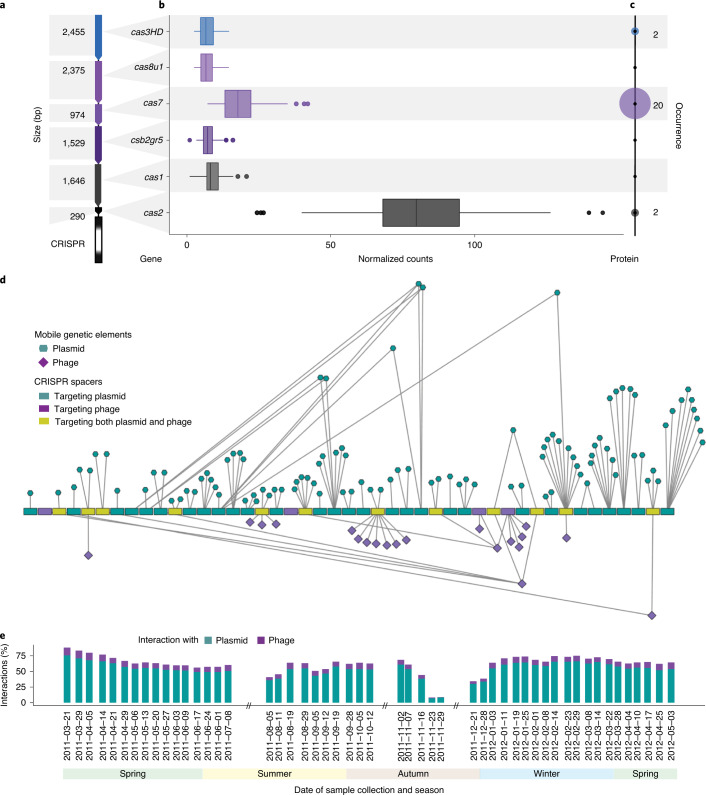
The CRISPR–Cas system of *M. parvicella*. **a**, The CRISPR–*cas* locus predicted within a reconstructed population-level genome (rMAG-165) identified as *M. parvicella*. **b**, MT-based expression levels of the corresponding *cas* genes. Boxplots represent expression levels aggregated from 51 time points based on normalized read counts. Data are presented as median values, Q1–1.5 × interquartile range (IQR) and Q3 + 1.5 × IQR. **c**, MP-level representation of Cas proteins. The numbers represent the number of time points at which at least one peptide of the corresponding Cas protein was detected. **d**, Representation of the active CRISPR spacers (gain or loss of spacer within the time series) assigned to *M. parvicella*. The order of the spacers is based on their first occurrence within the time series. **e**, Spacer-iMGE-based interactions represented per time point as percentages of the global interactions of *M. parvicella*.

To further understand the iMGE–host dynamics in relation to the maintenance of microbial populations of interest, we focused on the dominant population within the community, *M. parvicella*^15,37–39^, which constitutes ~30% of the community at specific dates (Fig. 1). More specifically, it showed distinct characteristics in the community and network dynamics, such that time points with decreased *M. parvicella* abundance exhibited a higher number of overall CRISPR-mediated interactions (Fig. 4 and Supplementary Videos 1 and 2), which was further supported by the negative correlations with the total number of plasmid–host interactions over time (*r* = −0.33, *P* = 0.017) and phage–host interactions over time (*r* = −0.40, *P* = 0.004). However, after focusing on the population-level CRISPR-based iMGE–host interactions of *M. parvicella*, we observed a positive correlation between the population abundance over time and its number of iMGE–host interactions, that is, plasmid–host (*r* = 0.63, *P* ≈ 0) and phage–host (*r* = 0.25, *P* = 0.02). Finally, the iMGE–*M. parvicella* network exhibited a highly modular structure, whereby a set of iMGEs interacted with its set of spacers (Fig. 4).

**Fig. 5:**
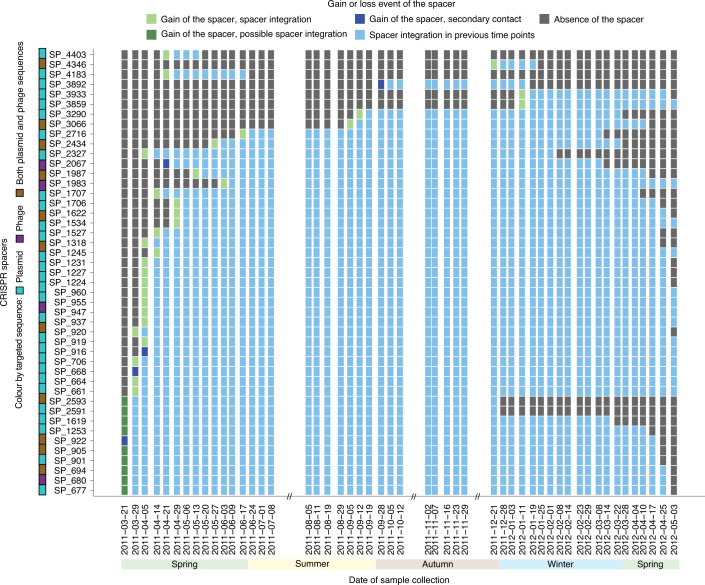
Spacer acquisition dynamics in the *M. parvicella* population. Dynamics of spacers assigned to the *M. parvicella* population. The *y* axis includes the spacer identities. The coloured boxes next to the spacer identities indicate the type of iMGE targeted by that spacer. The boxes within the plot are coloured based on the presence (light blue) or absence (dark grey) of the spacer within the CRISPR array for each time point. Green boxes represent spacer gain events, specifically light green for spacer integration (iMGE is detected before the spacer) and dark green for a putative spacer integration event (iMGE and spacer are detected at the same time point). Dark blue boxes represent potential secondary contact events (spacer detected before the iMGE).

**Extended Data Fig. 8:**
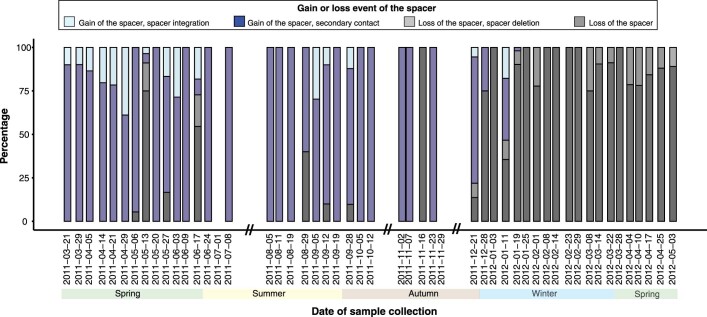
Spacer acquisition dynamics in *Candidatus* Microthrix parvicella population. Barplot representing the percentage of spacers per time-point reflecting a gain or loss events. Gain events are defined as: i) “Gain of the spacer, spacer integration” when the iMGE was detected before or at the same timepoint as its linked spacer, and ii) “Gain of the spacer, secondary contact” when the spacer was detected before the linked iMGE within the time-series. Loss events are defined as: i) “Loss of the spacer, spacer deletion” when both the spacer and the iMGE are not detected anymore within the remainder of the time-series, and ii) “Loss of the spacer” when the spacer is not detected within the time-series anymore but the iMGE is still detected after spacer loss. The labels on the x-axis indicate the sampling dates and the double slashes (//) on the time axis represent absence of samples from the sampled system.

**Extended Data Fig. 9:**
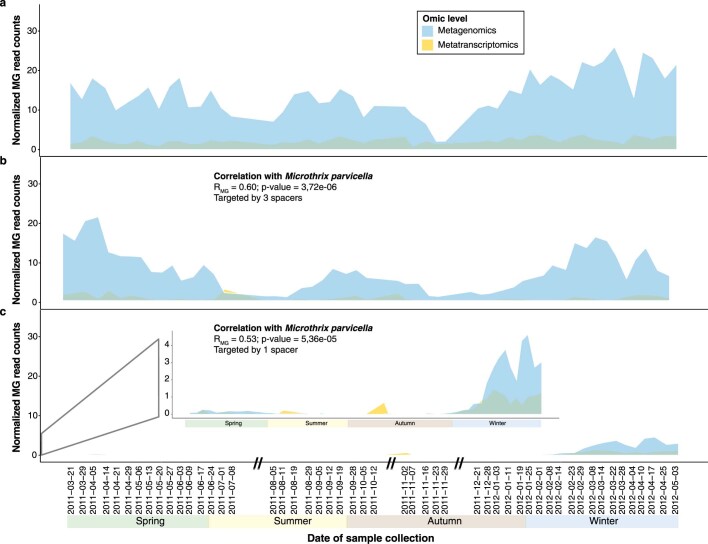
Abundance of *M. parvicella* and selected plasmid sequences targeted by the spacers of the same species. **a**, Metagenomics (MG)-based and metatranscriptomics-based (MT) abundance of *M. parvicella* over time. **b**, Abundance of plasmid contig “D28_L2.21_contig_56858”, with a size of 2,503 bps which is targeted by three spacers within *M. parvicella*’s CRISPR locus. **c**, Abundance of plasmid contig “D48_E1.25_contig_355826”, with a size of16,151 bps which is targeted by one spacer within *M. parvicella*’s CRISPR locus. Statistical tests were two-sided and adjusted for multiple comparisons.

We identified a single contig of 10,224 base pairs in length that encoded a complete CRISPR operon^40^. This contig shared 97.62% sequence identity with ‘*Candidatus* Microthrix parvicella Bio17-1’^37^ (Supplementary Note 9). Briefly, the contig contained 6 *cas* genes and 11 CRISPR repeats. Using the MT and MP data, we found that the *cas* genes within the rMAG were expressed over time, with *Cas2* showing the highest level of gene expression while *Cas7* was found more frequently at the protein level (Fig. 4). We were able to link a total of 670 spacers across the entire time series to this specific CRISPR locus. These spacers were present within an average of 25.5 time points (median = 28.5, s.d. = 14). Out of all the associated spacers, 433 lacked matches within the time series and 246 could be linked to a protospacer in at least one time point. Among these, 64 targeted plasmids, 24 targeted phages and 12 targeted both plasmids and phages (Fig. 4). Ten out of the 12 spacers targeting both had matches in protein-coding genes, including sigma 70 factor of RNA polymerase, GDSL-like lipase 2 and helix-turn-helix domain 23, which are genes known to be widely encoded by both plasmids and phages. Additionally, we inspected the activity of spacers within the CRISPR loci and observed 45 spacers with gain or loss events (Fig. 5). Similar to the community level, there was also a shift in gain to loss events occurring after the community shift on 2011-12-28 (Extended Data Fig. 8). Overall, the *cas* gene and Cas protein expression levels, coupled to spacer dynamics targeting more plasmids (example shown in Extended Data Fig. 9) than phages, demonstrate a highly active CRISPR–Cas system within *M. parvicella*.

**Extended Data Fig. 10:**
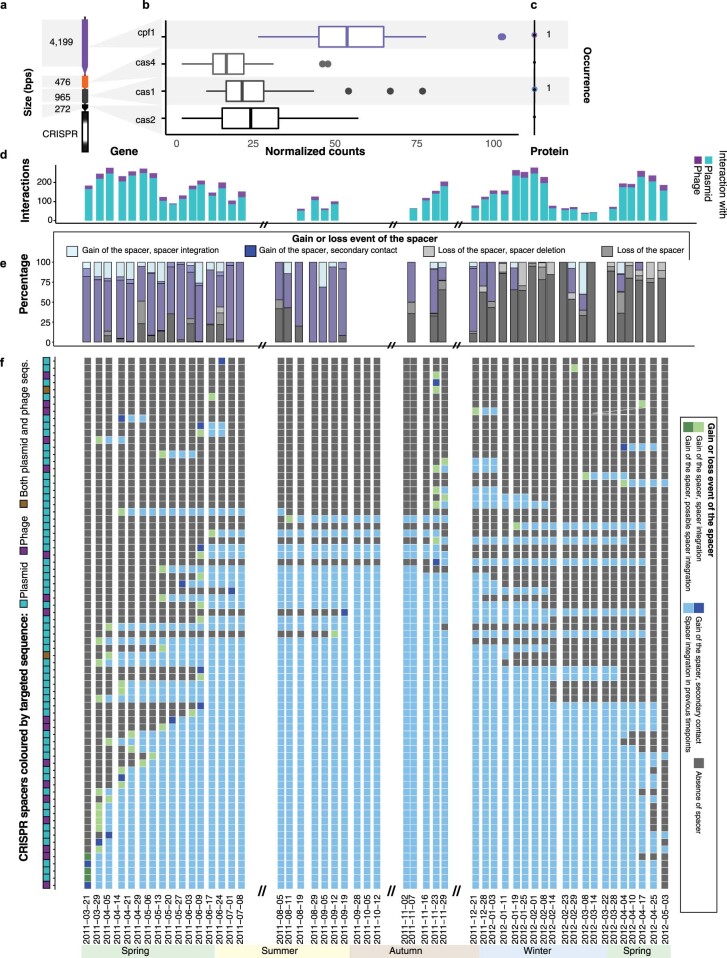
Spacers acquisition dynamics of the rMAG-40 population classified as *Leptospira biflexi*. **a**, CRISPR-Cas operon. **b**, Metatranscriptomics-based expression levels of the cas genes. Boxplot represents expression levels aggregated from 51 timepoints based on normalized read counts. Data are presented as median values, Q1–1.5 x IQR and Q3 + 1.5 x IQR. **c**, Metaproteomic-level representation of Cas proteins. The numbers represent the number of time points where at least one peptide of the Cas protein was detected. **d**, Barplot representing the number of interactions between rMAG-40 and iMGEs. The purple section of the bars represent the number of interactions with phages, while in turquoise represent interactions with plasmids. **e**, Barplot representing the percentage of spacers per time-point with a gain or loss event. Gain events are defined as: i) “Gain of the spacer, spacer integration”, when the iMGE was detected before, or at the same timepoint, as its linked spacer, and ii) “Gain of the spacer, secondary contact”, when the spacer was detected before the linked iMGE, within the time-series. Loss events are defined as: i) “Loss of the spacer, spacer deletion”, when both the spacer and the iMGE are not detected anymore within the rest of the time-series, and ii) “Loss of the spacer”, when the spacer is not detected within the time-series anymore, but the iMGE is still detected after spacer loss. **f**, Dynamics of spacers assigned to the rMAG. The y-axis shows the IDs of spacers assigned to the rMAG. The labels on the x-axis indicate the sampling dates and the double slashes (//) on the time axis represent absence of samples in the sampled system. The labels on the x-axis indicate the sampling dates and the double slashes (//) on the time axis represent absence of samples in the sampled system.

In contrast to *M. parvicella*, other populations exhibited more dynamic CRISPR loci, such as the rMAG-40 classified as *L*. *biflexa*, and less dynamic loci, such as the rMAG-31 classified as *Intrasporangium calvum* (Supplementary Note 10). *L. biflexa* has eight putative CRISPR loci and a locus of *cas* genes classified as type V (Supplementary Table 16 and Extended Data Fig. 10), and these contained a total of 680 spacers, of which 146 exhibited gain or loss within the time series. The population with the highest amount of spacers was rMAG-73, which was classified as *C*. *pinensis*, with CRISPR type III and a total of 1,119 spacers, of which 306 were active (that is, with either gain or loss events). Overall, the size of the CRISPR locus did not directly relate to spacer gain or loss. Finally, we observed that different population-level CRISPR–Cas dynamics exist at the level of gene and protein expression as well as spacer integration activity. Based on our results, *M. parvicella* populations contain a functional CRISPR system, but use it sparingly compared with other populations.

## Discussion

We presented an extensive time-resolved, integrated meta-omics analysis of CRISPR-mediated iMGE–host interactions. Given the vast extent of unresolved bacterial taxa as well as plasmid and phage sequences in this community, the reliance on existing sequence databases would have greatly limited the analysis of key community members. Our reference-independent approach, including de novo genomic assembly, binning and plasmid/phage prediction, were required to analyse this dataset. We were able to link microbial population genomes (rMAGS) to iMGEs using spacer–protospacer links^24^, unlike previous approaches that have relied on abundance levels^41^. Overall, our approach of resolving interaction dynamics between iMGEs and their hosts revealed an enrichment in CRISPR-based plasmid targeting relative to phages.

To extract coherent information across the time series, we minimized redundancy concerning population-level genomes, CRISPR information and iMGEs. The aforementioned procedures may potentially result in a dilution of information, especially regarding underlying species- and strain-level diversity. However, this trade-off was necessary considering the inherent properties of the time-series dataset, namely, in relation to the appearance, disappearance and/or reappearance of features over time. More importantly, our stringent methodology allowed us to balance the advantages of a de novo assembly-based approach, that is, detecting novel microbial and iMGE populations, while enabling us to track the populations over time.

We systematically optimized the plasmid and phage prediction process by applying an ensemble approach to reduce bias stemming from a single tool, establishing associations of iMGEs and specific rMAGs through binning, identifying strong correlations between iMGEs and their associated rMAGs and using spacer–protospacer links to establish empirical evidence of interactions between rMAGs and iMGEs. Despite this, several limitations must be addressed, including the inherent inaccuracies of the plasmid and phage prediction tools, the inability to predict prophages within community and the lack of reliable taxonomic classifications of iMGEs.

Our ensemble approach for iMGE identification demonstrated that plasmids are highly abundant within the community. The stepwise linear modelling approach demonstrated that plasmids have a more pronounced impact on the dominant Microthrixaceae compared to phages. Furthermore, based on the extracted protospacer information, plasmids are targeted more often than their phage counterparts by CRISPR systems. In contrast to previous studies focused on CRISPR-mediated immunity against phages, our results support the notion that plasmids also play key roles in the adaptation and promotion of diversity^42^. In this context, BWWTPs are thought to be hotspots for the spread of ARGs through iMGEs^3,43^. Our data revealed a comparatively small fraction of plasmids encoding ARGs that are targeted by CRISPR systems, which suggests that bacteria retain potentially beneficial plasmids^44^, for example, those encoding ARGs^45^, but further detailed investigation including data from longer-term time series is required.

The period with decreased Microthrixaceae abundance (from 2011-11-02 to 2012-01-25) coincided with the increased in abundance of other families (for example, Leptospiraceae or Moraxellaceae), their corresponding plasmids and overall CRISPR-mediated interactions. Based on this information, the increase in plasmids suggests a short-term fitness advantage for Leptospiraceae and Moraxellaceae populations, on the one hand. On the other hand, CRISPR-mediated links indicated CRISPR-based suppression of those plasmids in a possible drive towards the normalization of community structure and function, including the dominance of *M. parvicella*. However, any direct cause–effect relationships remain to be further explored under controlled laboratory conditions.

In relation to phages, we found that they tended to correlate with specific families, for example, Moraxellaceae and Leptospiraceae, which exhibited acyclical dynamics in relation to the Microthrixaceae family, but showed a smaller effect in the linear models. Additionally, rMAG populations within the Moraxellaceae and Leptospiraceae families exhibited higher CRISPR activity in terms of phage-linked spacer gain or loss. In that regard, phages are known to affect specific populations, which, according to our data, does not include the dominant *M. parvicella*, as previously observed^46^. Therefore, future studies need to be directed towards deciphering the roles of individual plasmids and phages on specific populations, as well as the community as a whole.

Based on our observations, a strong case can be made to include iMGEs and CRISPR-based interactions as additional features into models that incorporate abiotic parameters (for example, temperature, pH and oxygen concentration) and biotic drivers (for example, population dynamics and inter-microbial population interactions)^41,47,48^, especially when such information can be extracted from MG data. The inclusion of such additional features may provide a more comprehensive model of community dynamics and process performance.

Finally, the composition of CRISPR loci is highly environment-specific^49^, which should translate into environment-specific CRISPR-mediated interactions. Therefore, the present study should be repeated on samples from other environments to provide a broader understanding of CRISPR-based interactions in relation to iMGEs^50^.

## Methods

### Sampling

Individual floating sludge islets within the anoxic tank of the Schifflange BWWT plant (Esch-sur-Alzette, Luxembourg; 49° 30′ 48.29″ N; 6° 1′ 4.53″ E) were sampled according to previously described protocols^15^. Samples are indicated as dates (YYYY-MM-DD). Time-resolved sampling included two initial sampling dates (2010-10-04 and 2011-01-25) as previously reported^15,48^. More frequent sampling was performed from 2011-03-21 to 2012-05-03, of which data from three samples (2011-10-05, 2011-10-05 and 2012-01-11) have been previously published^15^.

### Concomitant biomolecular extraction and high-throughput meta-omics

Concomitant biomolecular extraction of DNA, RNA and proteins as well as high-throughput measurements to obtain MG, MT and MP data were carried out according to previously established protocols^15,48,51^.

### Isolate culture, genome sequencing and assembly

A total of 85 isolate cultures of lipid-accumulating bacterial strains were derived from the sludge islets sampled from the same anoxic tank described above. The isolation protocol, including screening for lipid-accumulation properties (via Nile Red staining), DNA extraction and sequencing, was performed as previously described^48,51^. The genomic data were assembled and analysed using an automated version of a previously described workflow^51^ that spanned sequencing read preprocessing, de novo assembly and gene annotation (see the section “Code availability”). The genome of ‘*Candidatus* M. parvicella Bio17-1’ was obtained from the publicly available NCBI BioProject database PRJNA174686 (ref. ^37^).

### Co-assembly of MG and MT data

Sample-wise integrated MG and MT data analyses were performed using IMP^21^ (v.1.3) with the following customized parameters: (1) Illumina Truseq2 adapters were trimmed; (2) the step involving the filtering of reads of human origin step was omitted for preprocessing; and (3) the MEGAHIT de novo assembler^52^ was used for the co-assembly of MG and MT data. Nonpareil2 (ref. ^53^) was applied to the preprocessed MG and MT data to assess the relative depth of coverage.

### MP analyses

Raw mass spectrometry files were converted to MGF format using MSconvert with default parameters. The resulting files were used to run the Graph2Pro pipeline^54^ together with the corresponding assembly graphs from MEGAHIT, which allowed the integration of MG, MT and MP data. Assemblies often result in fragmented consensus contigs, thus leading to a loss of information on strain variation and to open-reading frames spanning multiple contigs. The Graph2Pro pipeline combines the Graph2Pep algorithm and FragGeneScan^55^ to predict peptides from short and long edges of the graph even if the peptides span multiple edges. Graph2Pro further predicts protein sequences from the graphs of the IMP-based co-assemblies using identified peptides as constraints. To produce the final protein identifications, MP data were searched against the sample-specific databases derived from Graph2Pro.

The combined set of tryptic peptides was used as the target database for peptide identification using the MS-GF+ search engine^56^ and customized parameters. The instrument type was set to a high-resolution LTQ with a precursor mass tolerance of 15 ppm and an isotope error range of −1 and 2. The minimum and the maximum precursor charges were set to 1 and 7, respectively. The false discovery rate (FDR) was estimated by using a target-decoy search approach, whereby reverse sequences of the protein entries were generated while preserving the carboxy-terminal residues (KR) and concatenated to the database. All identifications were filtered to achieve an FDR of 1%.

Identified peptides from the Graph2Pro pipeline were assigned using peptidematch^57^ against Prokka-based^58^ predictions from IMP for protein-coding sequences of the rMAGs, and prodigal-based predictions^59^, including fragmented genes (see section “Gene annotation of phage- and plasmid-derived contigs” below) for protein-coding sequences of the iMGEs.

### Binning, selection of representative genome bins, taxonomy and estimation of abundance

Co-assembled contigs from each time point were binned as previously described^60^. Binning was based on nucleotide signatures, presence of single-copy essential genes and MG depth of coverage. Bins from each time point with at least 28% completeness and contamination of less than 20% along with the 85 isolate genomes were subjected to a dereplication process using dRep^22^ (v.0.5.4) to select rMAGs. Accordingly, the following dRep parameters were set: (1) genome completeness of 0.6 (based on CheckM^61^ (v1.0.7)); (2) strain heterogeneity of 101; (3) average nucleotide identity (ANI) threshold of 0.6 to form primary clusters; and (4) ANI threshold of 0.965 to form secondary clusters. Taxonomic classification was performed using a customized version^62^ of AMPHORA2 (ref. ^63^). Additionally, taxonomic classification was performed using sourmash^64^ 2.0.0a1-lca-version with a kmer-length of 21 and a threshold of 4 using an existing database that included around 87,000 microbial genomes (downloaded on 09 November 2017 from https://osf.io/s3jx8/download).

AMPHORA2-based predictions for individual marker genes were combined via the summation of the associated assignment probabilities. If the summed probability scores for the highest-scoring taxonomic level constituted less than one-third of the total probability scores, the assignment was discarded as a ‘low confidence assignment’. Taxonomic assignments of AMPHORA2 and sourmash-lca were combined and then filtered to select a final taxonomic assignment for the rMAGs, giving priority to predictions from sourmash-lca due to higher expected specificity and an updated database. We then selected rMAGs with a ‘completeness − contamination’ value of ≥50% for further downstream analyses.

To represent population-level abundance and transcription levels, the preprocessed MG and MT paired- and single-end reads from all the time-series samples were mapped onto the collection of rMAGs using bwa mem^65^, and contig-level average depth-of-coverage values were extracted for the MG and MT data. Gene-level MT read counts for all the predicted genes present within each rMAG were normalized using R statistical software to obtain the corresponding gene expression values.

### Identification of CRISPR elements

CRISPR information (that is, spacers, repeats and flanking sequences) were predicted using CRASS^66^ (v.0.3.8) based on the IMP-based preprocessed MG and MT paired- and single-end reads as input. MetaCRT^67^ was used to predict spacers and repeats from IMP-based MT and co-assembled contigs. A custom script was used to extract flanking regions from the metaCRT results.

The redundancy of spacers, repeats and flanking sequences was reduced by clustering the sequences with CD-HIT-EST^68^ (v.4.6.7). Spacers were clustered using 90% sequence identity^69,70^, covering the entire length of the compared sequences^69^. CRISPR-flanking regions were clustered using 99% sequence identity, with at least 97.5% coverage of both the compared sequences. Conversely, the CD-HIT-EST clustering parameters for repeats were manually determined by clustering the known repeats belonging to a single CRISPR locus of ‘*Candidatus* M. parvicella Bio17-1’^37^. Specifically, the sequence identity parameter was first set to 99% and the sequence coverage was set to 100%. These parameters were reduced by 5% in the subsequent iterations until all repeats were regrouped into a single cluster. Next, all the known repeats of *M. parvicella* were clustered at 80% sequence identity, covering the length of at least 75% of the shorter sequence. These parameters were used for the clustering of all repeats. FASTA headers of all the sequences were left unchanged (that is, *-d* parameter in CD-HIT-EST) because they contained information required for downstream analyses (for example, sample name, contig name and CRASS-computed coverage). The clustering procedure for the different CRISPR elements yielded non-redundant sequences of repeats, spacers and flanking regions.

Spacer abundance values were estimated by extracting their coverage values from CRASS. Equivalent information was obtained from metaCRT by using bwa-mem to map MG and MT reads from each of the time-resolved samples to the entire set of contigs predicted by metaCRT (that is, contigs containing at least one CRISPR locus). The depth-of-coverage information was derived using bedtools^71^. Based on this, abundance values were extracted for each of the predicted spacers per time point. The depth-of-coverage information of the metaCRT contigs was then consolidated using CRASS coverage results by referring to the non-redundant spacer clusters (derived from CD-HIT-EST). The consolidated results are hereafter referred as ‘spacer abundance values’. Specifically, the spacer abundance values from the specific time points were assigned to the non-redundant spacers, thereby allowing a temporal representation of spacer abundance values. Subsequently, the spacer abundance values were transformed to counts per million (c.p.m.)^72,73^ per sample, and non-redundant spacers that had at least one read count in at least one sample were selected and the c.p.m. values were calculated. Finally, to determine the presence/absence of a given spacer, a minimum cut-off value of c.p.m. = 1 was applied. Applying standard cut-offs (that is, above 3–5) caused loss of information from the short spacer sequences within the repetitive CRISPR regions, which usually do not recruit many reads during the mapping process.

### Linking rMAGs to CRISPR elements

The non-redundant flanking regions and repeats were used to associate MAGs with specific CRISPR loci using BLASTN^74^. Non-redundant CRISPR-flanking sequences and CRISPR repeats were searched against the contigs of the MAGs. Flanking sequences and MAG contig(s) exhibiting similarities of at least 95% identity and coverage of either (1) 80% for flanking sequences >100 bp or (2) 95% for flanking sequences <100 bp were retained for the downstream filtering steps. Next, the aforementioned flanking sequences for which the associated repeats had at least 75% identity and 80% coverage against the MAG contig(s) were further retained for downstream processing. After defining the selected flanking repeat sequences linked to a MAG, spacers linked to the repeat flanking sequences were then associated to the MAG. In this way, the composition of spacers per MAG was determined. Finally, all the CRISPR information belonging to a MAG was linked to its rMAG to preserve the maximum amount of CRISPR information.

CRISPR types and subtypes and *cas* genes were predicted from all the assembled contigs using CRISPRone^23^. The *cas* genes and CRISPR types were then assigned to their respective MAGs.

We then selected rMAGs predicted as *M. parvicella* (see the section “Binning, selection of representative genome bins, taxonomy and estimation of abundance”) to inspect the *cas* genes and CRISPR-type predictions. Next, we used CRISPRCasFinder^75^ to further confirm the selected *cas* genes and CRISPR-type predictions of *M. parvicella*. We performed manual curation on all the rMAGs predicted as *M. parvicella*. We identified a contig (D47_L1.43.1_contig_476300) of 10,224 bp that encoded a complete CRISPR operon that was highly similar to the CRISPR operon of the isolate genome of ‘*Candidatus* M. parvicella Bio17-1’. This contig was incorporated with rMAG-165.

### Identification of protospacers and protospacer-containing contigs

A BLASTN^74^ search was performed using all non-redundant spacers as queries against the contigs from all time points using the parameters defined in CRISPRtarget^76^. Spacer matches with at least 95% coverage and 95% identity were selected for further analysis^32^. Any IMP-based MT results or co-assembled contigs containing repeat sequences and/or identified by metaCRT to encode CRISPR sequences were excluded from downstream analyses. Accordingly, the remaining spacer matches (or complements) were defined as protospacers, and the respective contigs that contained at least one protospacer were defined as PSCCs and were retained as iMGEs.

### Classification of iMGEs

Bacteriophage sequences were predicted by analysing all co-assembled contigs using VirSorter^77^ (v.1.0.3) and VirFinder^78^ (v.1.0.0). Similarly, plasmid sequences were predicted using cBar^79^ (v.1.2) and PlasFlow^80^ (v.1.0.7). The predictions were consolidated by annotating candidate iMGE sequences as follows: ‘plasmid’ if the sequences were positively predicted by cBar and/or PlasFlow; ‘phage’ if the sequences were positively predicted by VirSorter and/or VirFinder; ‘ambiguous’ if the sequences were predicted as both plasmid and phage by any combination of the aforementioned tools; and, finally, ‘unclassified’ if they contained at least one protospacer and were not annotated as phage or as plasmid. Following this step, all iMGEs (that is, phages, plasmids, ambiguous and unclassified) were clustered using CD-HIT-EST with clustering parameters of 80% identity and at least 50% coverage, generating the non-redundant set of iMGEs. The classification/annotation of representative clusters was retained for the downstream analyses. Finally, BLASTN^74^ was performed on the clustered contigs against NCBI plasmid and virus databases to retrieve their taxonomy.

Genomic and transcriptomic abundances of the iMGEs were obtained by mapping the IMP-preprocessed MG and MT paired- and single-end reads from all time points to the iMGE representative contigs using bwa-mem^65^. The contig-level average depth of coverage derived from the MG and MT data represented the iMGE abundance and iMGE gene expression, respectively.

### Gene annotation of phage- and plasmid-derived contigs

Open reading frames within iMGEs were predicted using Prodigal^59^ (v.2.6) with the “meta” and “incomplete gene” settings. Predicted genes were annotated using hmmsearch^81^ against an in-house licensed version of the KEGG database^82^. KEGG function identifiers were then converted to the higher-level COG functional categories^83^. Finally, ARGs were annotated using hmmsearch against ResFam’s full HMM database^84^.

### Linear model of community dynamics

Correlations of family-level groups, whereby plasmids and phages were assigned to bacterial families based on their previous contig assignments to MAGs, were calculated using the “rcorr” function within the Hmisc R package. Euclidean distances of the correlation vectors were calculated using the “dist” function (stats R package). Next, hierarchical clustering was applied on the calculated Euclidean distances, using the “hclust” function (stats R package). The tree was then cut with a height parameter of four (that is, *H* = 4), using the “cutree” function from R stats package^85^.

The “lm” function from the R stats package was used to generate the models. To avoid overfitting, we restricted the linear models to a maximum of 15 family-level groups. Random sampling was performed for 100,000 model realizations, and model quality was assessed using the adjusted *R*^2^ value. In our first approach, we did not restrict the model composition and allowed all combinations with the same probability. Then, from the random sampling data, we ranked models based on the adjusted *R*^2^ value and looked for enrichments in specific families in the best models (*N* = 25, 50, 100). In the first iteration, we selected enriched families and iMGEs (that is, plasmids and phages) to obtain a global model, and then we selected the significant groups from the global model to obtain a reduced model. Once we had the models for the entire time series and the shorter-time intervals, we identified the common significant groups in all the models. Next, we removed the group Microthrixaceae plasmids from the reduced models for each time interval to assess the influence of these plasmids within the performance of the model.

### Network analyses and visualization

CRISPR-based plasmid–host and phage–host networks were defined by the co-occurrence of rMAGs, spacers and a targeted iMGE in at least one time point. Thus, if a given non-redundant spacer was assigned to a specific rMAG and this specific rMAG did not co-occur in at least one time point, this spacer was deemed inactive within this rMAG throughout the time series. Consequently, a spacer was assigned to a rMAG if, and only if, the spacer co-occurred with its assigned rMAG in at least one time point. Thus, the iMGEs targeted by the spacers assigned to rMAGs were used to build the CRISPR-based plasmid–host and phage–host networks. Finally, the time-point-specific networks were built on the basis of the presence/absence of the rMAGs and their linked plasmids or phages.

Network properties such node degree, betweenness and closeness were estimated by the function “speciesLevel” within the bipartite R package^86^. Modularity, defined by the value of *Q*^87^, and nestedness, defined as the value of the nestedness matrix based on overlap and decreasing fill (NODF)^88^, were calculated using the functions “computeModules” and “nested”, respectively.

Visualization and manual inspection of the networks were performed using Cytoscape^89^ (v.3.6.1). R (v.3.4.1), together within the “tidyverse” framework, was used for processing data tables, statistical analyses and data visualization^90^.

### Estimation of spacer gain–loss and CRISPR locus dynamics

Based on the previously calculated c.p.m. per rMAG, their assigned spacers and iMGEs, the dates of the first and the last occurrence within the time series were defined. We subsequently defined events of gain and loss of spacers and possible secondary encounters of the iMGE with the rMAGs to resolve the variation within a given CRISPR array per population. These events were classified as follows: (1) gain of a given spacer if its first detection within the time series occurred after the first occurrence of its targeted iMGE; (2) probable gain of a given spacer if both the spacer and its targeted iMGE occurred for first time at the same time point; (3) probable secondary encounter if the spacer occurred for first time before its linked iMGE; (4) loss of a given spacer if last detection of the spacer occurred after the last detection of its linked iMGE; (5) probable loss of a given spacer if the last detection of both the spacer and the iMGE occurred at the same time point; (6) spacer loss before iMGE loss if the last occurrence of the spacer occurred before the last occurrence of the iMGE.

### Workflow automation

Bioinformatics workflow automation was achieved using Snakemake^91^ (v.3.10.2 to v.5.1.4).

### Computing platforms

All computing was run on the University of Luxembourg High-Performance Computing (ULHPC) platform^92^.

### Reporting Summary

Further information on research design is available in the Nature Research Reporting Summary linked to this article.

## Supplementary information

Supplementary Information Supplementary Figs. 1–8, Supplementary Table descriptions, Supplementary Tables 5, 6 and 8, Supplementary Video descriptions and Supplementary Notes 1–10.

Reporting Summary

Supplementary Video 1 Time-lapse plasmid–host network. Host nodes (circles) are coloured based on their taxonomy (see legend of Fig. 3).

Supplementary Video 2 Time-lapse phage–host network. Host nodes (circles) are coloured based on their taxonomy (see legend of Extended Data Fig. 7).

Supplementary Tables Supplementary Tables 1–4, 7 and 9–16.

Supplementary Data 1 Source Data for Supplementary Fig. 1: sample-wise summary, including sampling dates, MG and MT sequencing, large-scale bioinformatics processing from IMP, sample assessment results from Nonpareil and number of bins.

Supplementary Data 2 Source Data for Supplementary Fig. 2: number of predicted CRISPR elements, that is, spacers, repeats and flanking sequences per time point, by omic data (MG and MT) and by prediction tool.

Supplementary Data 3 Source Data for Supplementary Fig. 3: summary of reads mapping to the global set of the representative MAGs and iMGEs. Information was derived using ‘samtools flagstat’.

Supplementary Data 4 Source Data for Supplementary Fig. 4: Pearson correlation and *P* values between the 79 family-level groups of rMAGs and iMGEs, for the entire time series (*N* = 51 time points) and for the overlapping shorter-time intervals (*N* = 20 time points for each time interval).

## Data Availability

The genomic FASTQ files, rMAGs and isolate genomes from this work are publicly available within NCBI BioProject PRJNA230567. Similarly, MP data from this work are publicly available in the PRIDE database under the accession number PXD013655. Additional data are available via Zenodo (10.5281/zenodo.3774024 and 10.5281/zenodo.3766442). Additional publicly available projects cited by this work include NCBI BioProject PRJNA174686. Source data are provided with this paper.

## Source data

Source Data Fig. 1 MG-level relative abundance per sample, resolved at the family level of the rMAGs. Summary of CRISPR–Cas information per rMAG, including classification of the CRISPR–Cas system and taxonomic classification of the rMAGs. Source Data Fig. 2 Pearson correlation and *P* values between the 79 family-level groups of rMAGs and iMGEs for the entire time series (*N* = 51 time points). Source Data Fig. 3 Plasmid–host CRISPR-based network, including the presence (1) or absence (0) of an interaction per time point, and taxonomic classification of the rMAGs. Source Data Extended Data Fig. 1 Number of predicted CRISPR elements, that is, spacers, repeats and flanking sequences per time point, by omic data (MG and MT) and by prediction tool. Source Data Extended Data Fig. 7 Phage–host CRISPR-based network, including the presence (1) or absence (0) of an interaction per time point, and taxonomic classification of the rMAGs.

## Code availability

The code is available on three separate repositories: (1) the IMP, binning and population genomes can be found in https://github.com/shaman-narayanasamy/LAO-time-series (10.5281/zenodo.3988660); (2) the CRISPR and MGE analyses can be found in https://github.com/susmarb/LAO_multiomics_CRISPR_iMGEs (10.5281/zenodo.3988592); and (3) the isolate assembly analyses can be found in https://github.com/shaman-narayanasamy/Isolate_analysis (10.5281/zenodo.3988667).
