# Supplementary material for: Roles of bacteriophages, plasmids and CRISPR immunity in microbial community dynamics revealed using time-series integrated meta-omics
Source: Nat Microbiol. 2020 Nov 2;6(1):123–35. doi: 10.1038/s41564-020-00794-8 (PMC7752763; doi:10.1038/s41564-020-00794-8)
Supplement: Supplementary file 1 — Supplementary Figs. 1–8, Supplementary Table descriptions, Supplementary Tables 5, 6 and 8, Supplementary Video descriptions and Supplementary Notes 1–10. [file 41564_2020_794_MOESM1_ESM.pdf]

nature research
https://doi.org/10.1038/s41564-020-00794-8

## Supplementary information

# Roles of bacteriophages, plasmids and CRISPR immunity in microbial community dynamics revealed using time-series integrated meta-omics

In the format provided by the authors and unedited

# Supplementary information

## Roles of bacteriophages, plasmids and CRISPR-immunity in microbial community dynamics revealed using time-series integrated meta-omics

Susana Martínez Arbas†[1], Shaman Narayanasamy†[1,2], Malte Herold[1], Laura A. Lebrun[1], Michael R. Hoopmann[3], Sujun Li[4], Tony J. Lam[4], Benoît J. Kunath[1], Nathan D. Hicks[5,6], Cindy M. Liu[5,7], Lance B. Price[5,7], Cedric C. Laczny[1], John D. Gillece[5], James M. Schupp[5], Paul S. Keim[5,8], Robert L. Moritz[3], Karoline Faust[9], Haixu Tang[4], Yuzhen Ye[4], Alexander Skupin[1,10], Patrick May[1], Emilie E. L. Muller[1,11], Paul Wilmes*[1,12]

[1] Luxembourg Centre for Systems Biomedicine, University of Luxembourg, 7 Avenue des Hauts-Fourneaux, L-4362 Esch-sur-Alzette, Luxembourg.
[2] Current affiliation: Megeno S.A., 6A Avenue des Hauts-Fourneaux, L-4362 Esch-sur-Alzette, Luxembourg.
[3] Institute for Systems Biology, 401 Terry Avenue North, Seattle, Washington 98109, USA.
[4] School of Informatics, Computing and Engineering, Indiana University, 700 N. Woodlawn Avenue, Bloomington, IN 47405, USA.
[5] TGen North, 3051 West Shamrell Boulevard, Flagstaff, Arizona 86001, USA.
[6] Current affiliation: Harvard T.H. Chan, School of Public Health, 677 Huntington Avenue, Boston, MA 02115, USA.
[7] Current affiliation: Department of Environmental and Occupational Health, Miken Institute School of Public Health, George Washington University, 1918 F Street, NW, Washington, DC 20052, USA.
[8] The Pathogen and Microbiome Institute, Northern Arizona University, Flagstaff, AZ 86011-4073, USA.
[9] Laboratory of Molecular Bacteriology, KU Leuven, Herestraat 49, 3000 Leuven, Belgium.
[10] Department of Neuroscience, University of California, 9500 Gilman Drive, San Diego, La Jolla, CA 92093, USA.
[11] Department of Microbiology, Genomics and the Environment, UMR 7156 UNISTRA-CNRS, Université de Strasbourg, Strasbourg, France.
[12] Department of Life Sciences and Medicine, Faculty of Science Technology and Medicine, University of Luxembourg, 7 Avenue des Hauts-Fourneaux, L-4362 Esch-sur-Alzette, Luxembourg.
† These authors contributed equally to this work
*Corresponding author: paul.wilmes@uni.lu

This file contains supplementary figures, supplementary table legends, supplementary video legends, and supplementary notes, including additional relevant references.

# Supplementary Figures

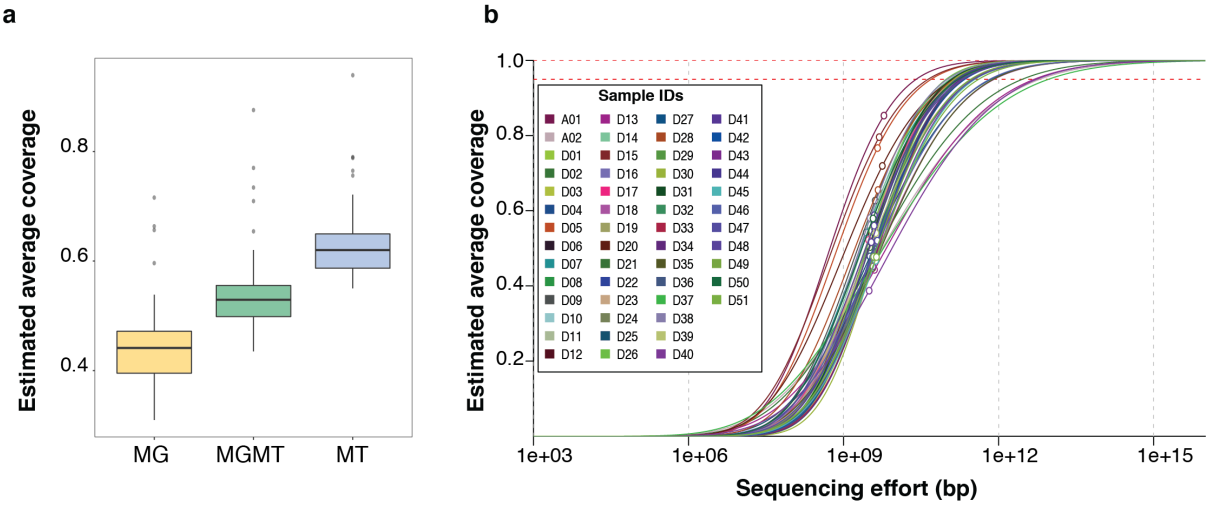


**Figure S1 | Sequencing depth assessment of the metagenomic (MG) and metatranscriptomic (MT) datasets. a**, Summary of Nonpareil[1,2] results based on IMP-based preprocessed reads within all samples in the time series and the two initial samples (n=53 *in situ* samples). Data are presented as median values, Q1 - 1.5 x IQR and Q3 + 1.5 x IQR. Evaluation was performed for each of the following data sets: i) MG, ii) MT, and iii) combined MG and MT (i.e. MGMT) reads. **b**, Each curve represents a Nonpareil coverage model for each sample, whereby the x-axis value corresponds to the upper plateau of the curve (i.e. dotted red lines where y-axis value ~1.0) and represents the theoretical sequencing effort required to cover all DNA and RNA elements within the community in terms of sequenced base pairs (bp). The circles on the curves represent the actual sequencing coverage of a given sample. The colors of the curves represent the individual samples. Supplementary Table 1 provides detailed information on Nonpareil analyses.

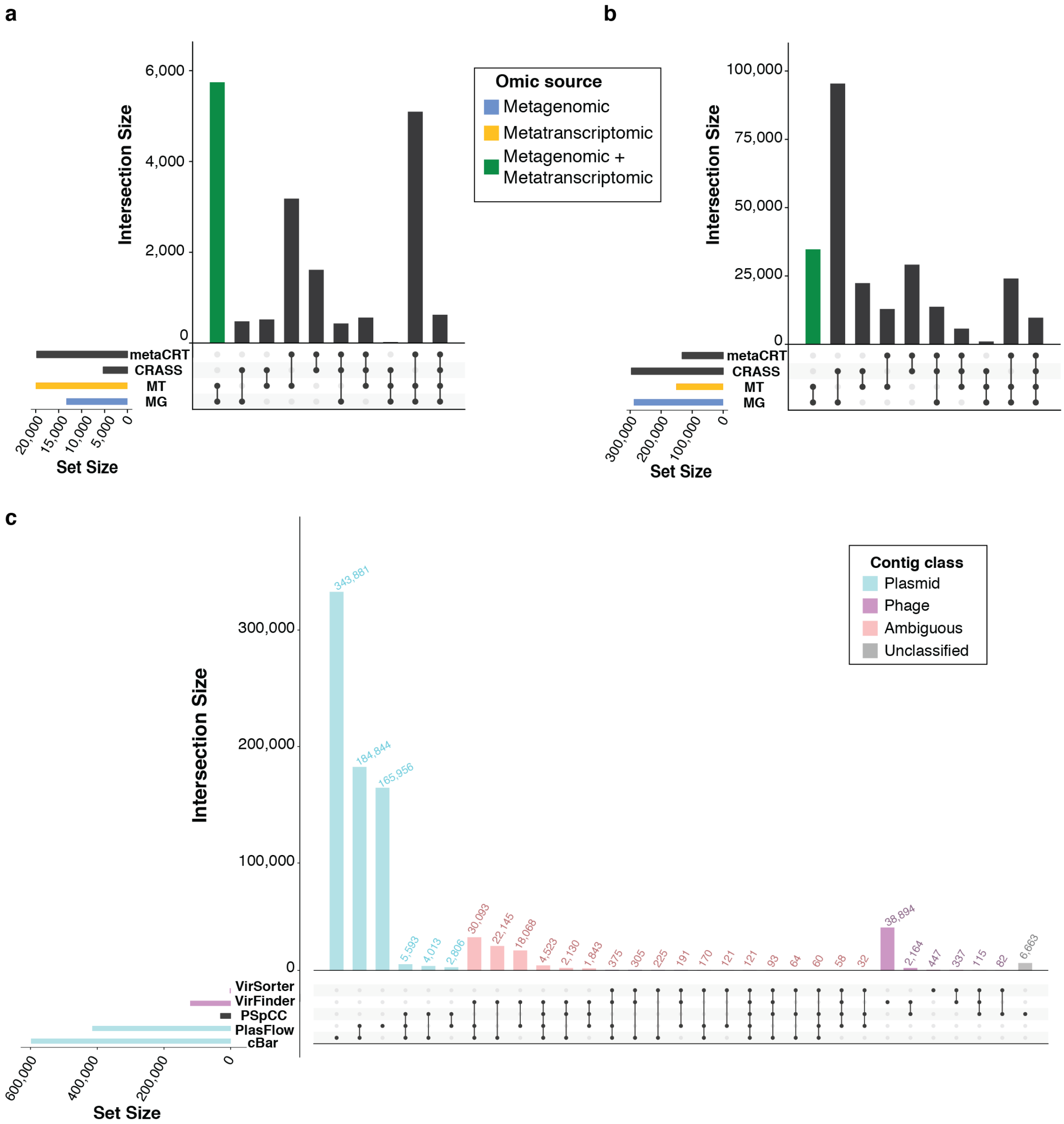


**Figure S2 | Prediction of CRISPR elements and invasive mobile genetic elements (iMGEs). a,** Upset plot representing the repeats predicted in the different omic levels and based on the different tools used. **b,** Upset plot representing the spacers predicted in the different omic levels and based on the different tools used. **a** & **b**, The vertical bars represent the intersection between the aforementioned omic levels and/or tools. c) Upset plot representing the prediction of iMGEs using different methods (horizontal bars) and their relevant intersections (vertical bars). The colours of the vertical bars represent the designated annotation after consolidating the prediction of all the different tools.

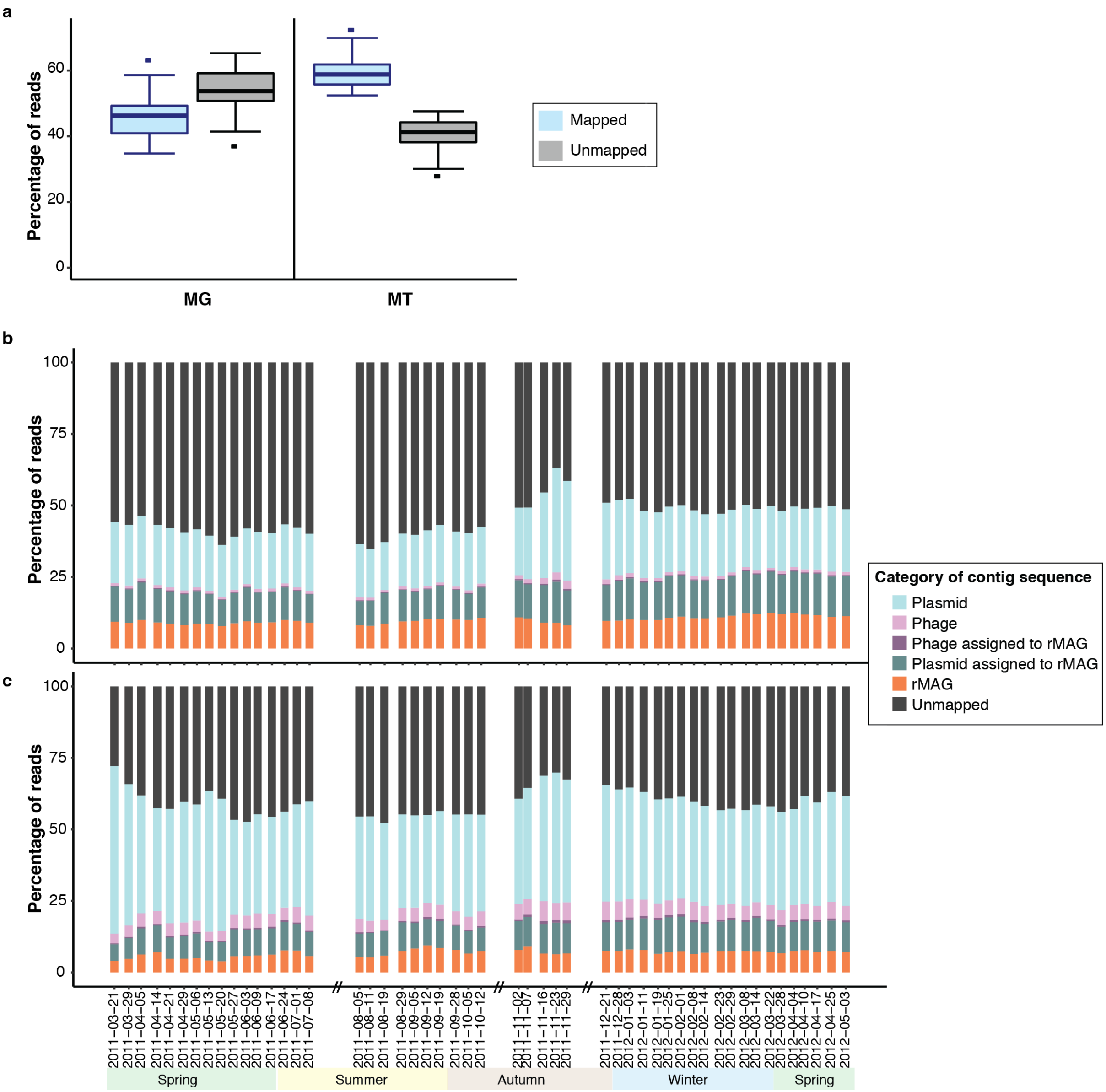


**Figure S3 | Mapping of the MG and MT reads against representative metagenomics assembled genomes (rMAGs) and invasive mobile genetic elements (iMGEs). a**, Boxplot representing the percentages of mapped and unmapped MG and MT reads within samples from the entire time-series (n=51 *in situ* samples). Data are presented as median values, Q1 - 1.5 x IQR and Q3 + 1.5 x IQR. **b**, Barplot representing the percentages of mapped and unmapped MG reads per time point. **c**, Barplot representing the percentages of mapped and unmapped MT reads per time point. The labels in the x-axis indicate the exact sampling dates, and the double slashes (//) on the time axis represent absence of samples.

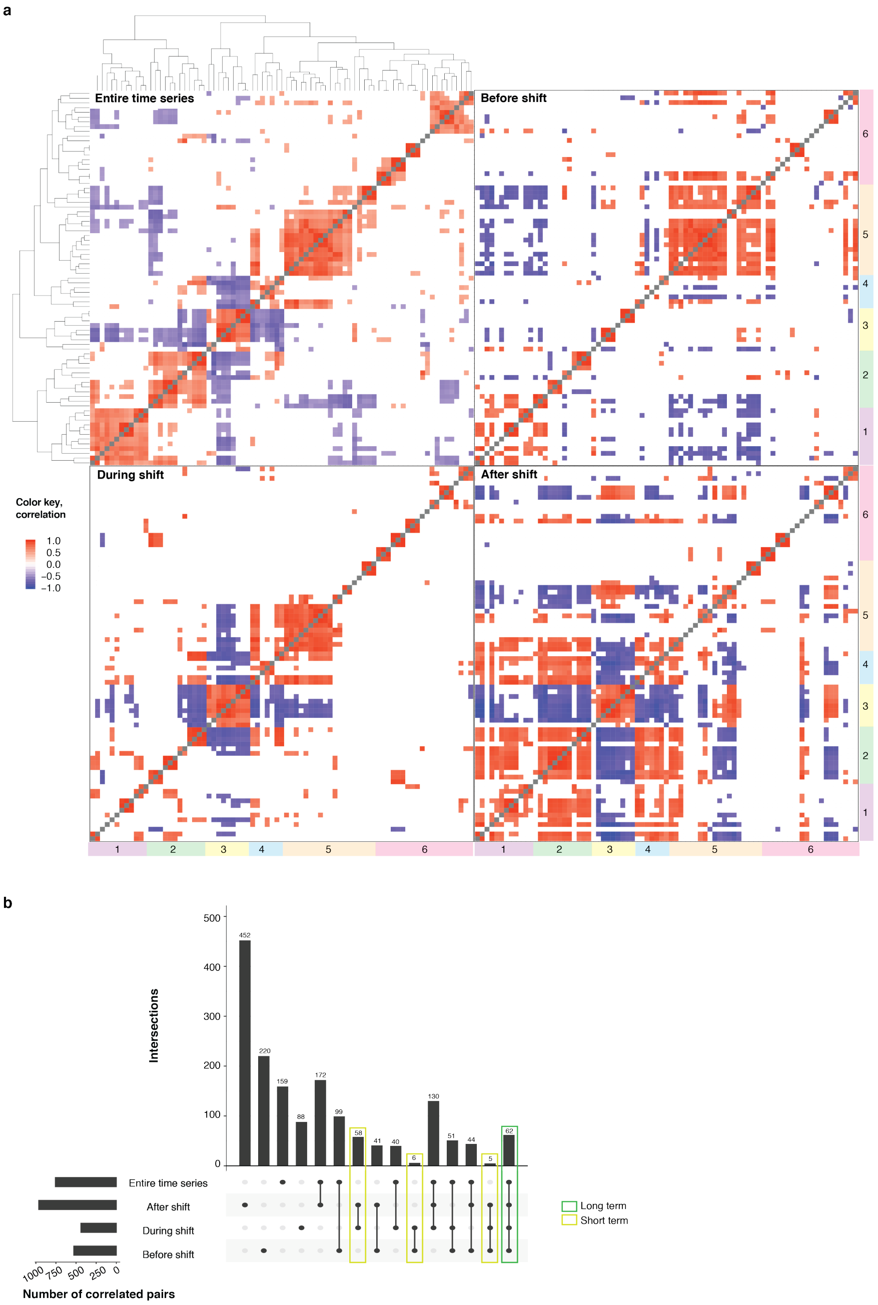


**Figure S4 | Correlation within longer- and shorter-term time intervals. a,** Correlation heatmaps based on entire time-series and shorter-term time intervals (defined in Fig. 2). The rows and columns of all heatmaps preserve the order of the hierarchical clustering from the entire time-series correlations. Similarly, the coloured strip annotations on the right and bottom of the heatmaps represent clusters 1-6 of Fig. 2. The values within the heatmaps represent significant (threshold: $p \leq 0.001$) correlations. Statistical tests were two-sided and adjusted for multiple comparisons. **b**, Upset plot represents the number of significant correlated pairs (from the heatmap) within the entire time-series and shorter-term intervals (horizontal bars). The number of intersections between those pairs in different time intervals (vertical bars). The coloured boxes represent the intersections that represent longer-(entire time-series) and shorter-term dynamics, respectively. Supplementary Table 10 provides detailed information on the common correlating pairs between the time intervals.

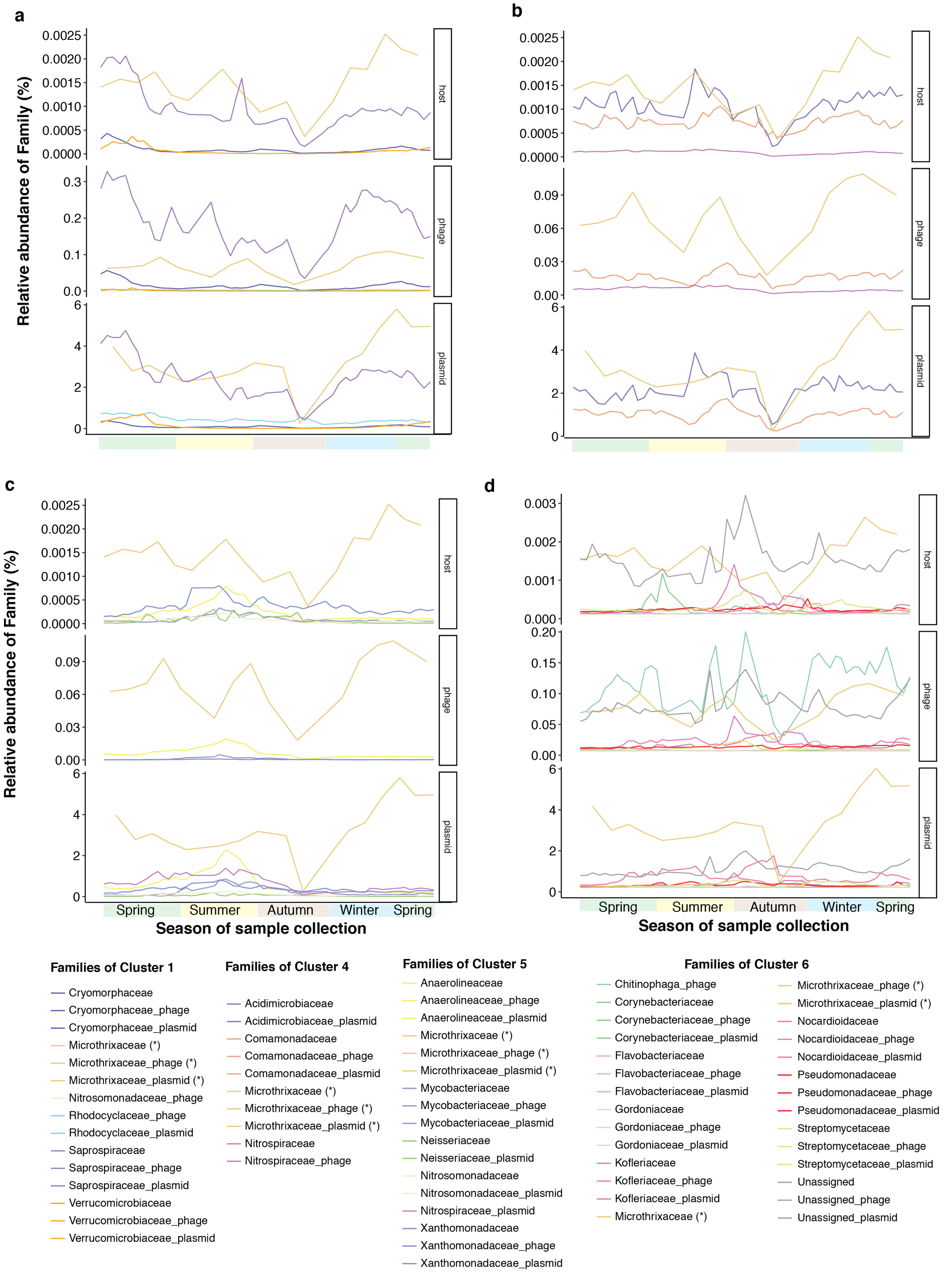


**Figure S5 | Dynamics of clusters comprised of bacterial-, plasmid- and phage- groups.** The rMAGs were grouped together at the family-level. Plasmids and phages were grouped based on their family-level association, i.e., binned together with an rMAG of a given family. The bacterial, plasmid and phage groups were clustered based on the correlation of their cumulative group-level abundance dynamics (Fig. 2). Figures representing the dynamics of the

groups within **a**, Cluster 1, **b**, Cluster 4, **c**, Cluster 5, **d**, Cluster 6. The *Microthrixaceae* family, and its associated plasmid and phage groups are found in Cluster 2 (shown in Extended Data Fig. 4). The plots display the abundance dynamics of the *Microthrixaceae* family, plasmid and phage groups as a point of reference (i.e., not part of those clusters). Therefore, those groups are marked with an asterix (*) within those figures. The group "Unassigned" represents the rMAGs that could not be classified on the family-level. Accordingly, the groups "Unassigned_plasmid" and "Unassigned_phage" represent plasmids and phages that were assigned to those rMAGs within the "Unassigned" group. Plasmids and phages that were not assigned to any rMAG (via binning) were omitted from all figures. Relative abundance values on the y-axis were derived from MG data. The x-axis represents time, colour coded by seasons as labelled in panels **c** and **d**. Please refer to Fig. 1 for the exact sampling dates.

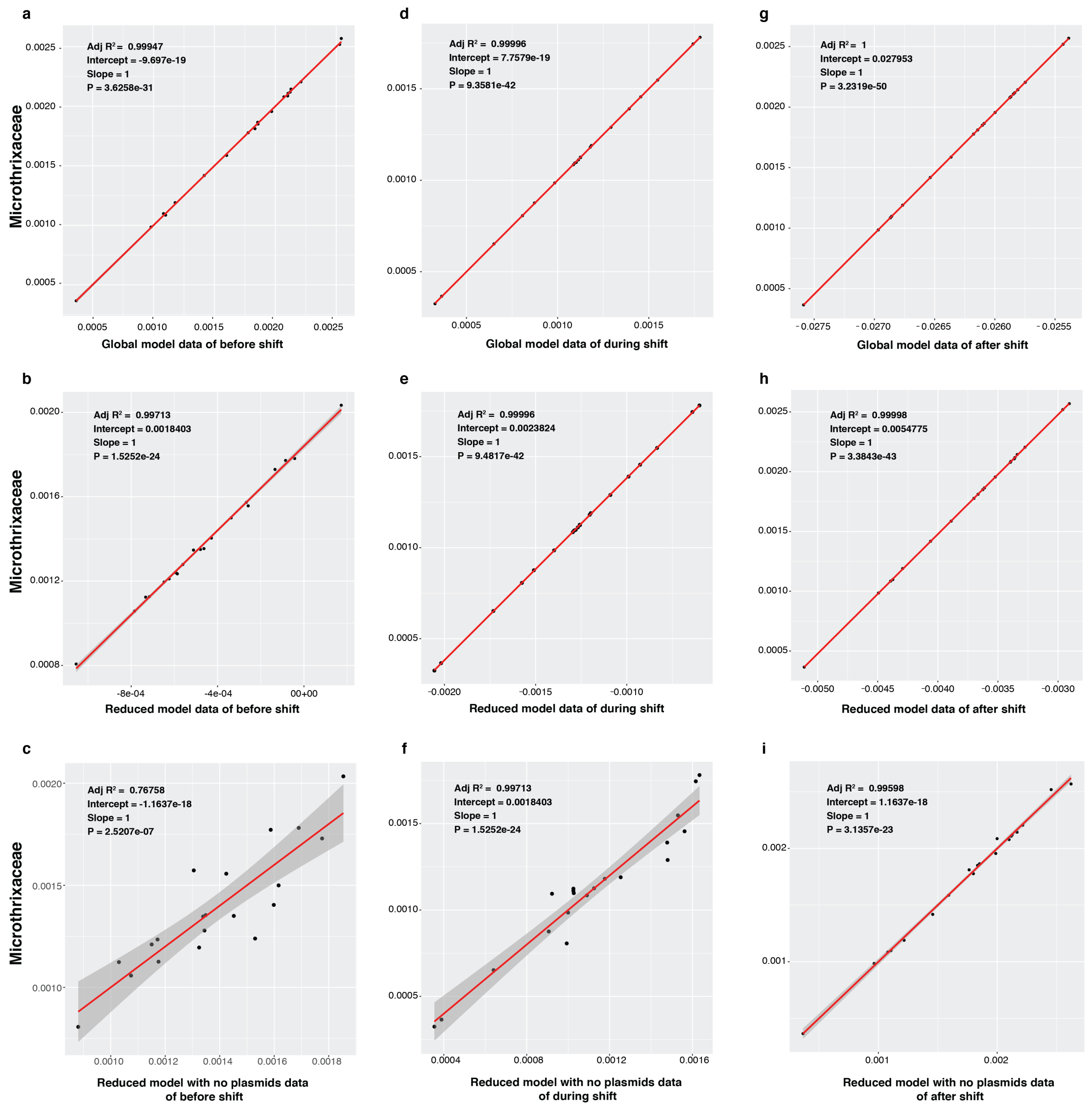


**Figure S6 | Linear models predicting *Microthrixaceae* family abundance within different time intervals.** Model data fitted to the raw data of the overlapping shorter-term intervals. All intervals consist of n=20 *in situ* samples, specifically **a, b, c,** Model data fitted to the raw data of the time interval before the community shift, between 2011-03-21 and 2011-08-29, specifically global, reduced and reduced without plasmids models, respectively. **d, e, f,** Model data fitted to the raw data of the time interval during the shift, between 2011-08-05 and 2011-01-19, specifically global, reduced and reduced without plasmids models, respectively, **g, h, i,** Model data fitted to the raw data of the time interval after the community shift, between 2011-12-21 and 2012-05-03, specifically global, reduced and reduced without plasmids models, respectively. Global linear models correspond to the optimal models in each time interval. Reduced linear models include only the significant features from the global models. Reduced models without plasmids include features from the reduced models but exclude plasmids of *Microthrixaceae*. Gray bands represent the +/- standard error measurement of the regression line. Statistical tests were two-sided and adjusted for multiple comparisons. Detailed results of the linear models, including model composition, residuals, and coefficients are shown in Supplementary Table 11.

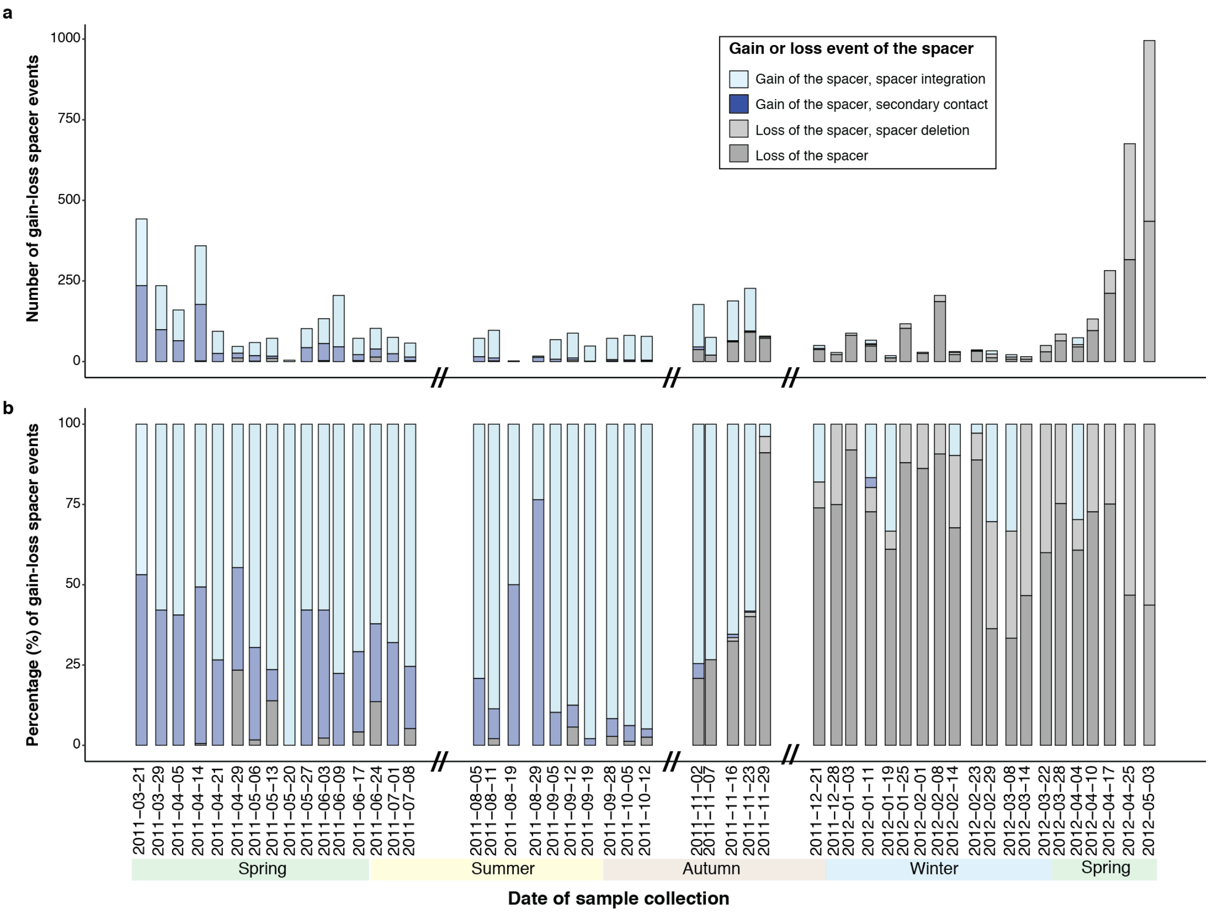


**Figure S7 | Gain and loss of CRISPR spacers targeting iMGEs**. **a**, Barplot representing the number of spacers per time-point reflecting a gain or loss event. **b,** Representation of **a** in percentages. Gain events are defined as: i) "Gain of the spacer, spacer integration", when the iMGE was detected before, or at the same timepoint, as its linked spacer, and ii) "Gain of the spacer, secondary contact", when the spacer was detected before the linked iMGE within the time-series. Loss events are defined as: i) "Loss of the spacer, spacer deletion", when both the spacer and the iMGE are not detected anymore within the rest of the time-series, and ii) "Loss of the spacer", when the spacer is not detected within the time-series anymore, but the iMGE is still detected after spacer loss. The labels on the x-axis indicate the sampling dates and the double slashes (//) on the time axis represent absence of samples in the sampled system.

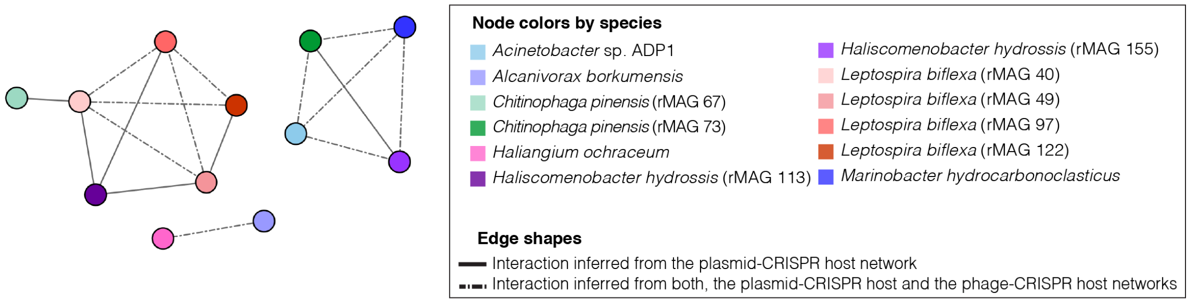


**Figure S8 | One-mode projection of the bipartite iMGE-CRISPR host networks**. Interaction network between microbial populations, where the interactions are inferred from the iMGE-CRISPR host interactions.

## Supplementary Tables

**Supplementary Table 1. Sample-wise summary**. This table includes sampling dates, MG and MT sequencing information, large-scale bioinformatics processing from IMP, sample assessment results from Non-pareil and number of bins.

**Supplementary Table 2. Metaproteomics summary**. Overview of the metaproteomic data per sample including the number of MS/MS spectra measured, number of spectra assigned to a peptide, the percentage of identified spectra and the total number of peptides.

**Supplementary Table 3. Taxonomy of rMAGs.** Summary of representative metagenome-assembled genomes (rMAGs), including standard assembly statistics and taxonomic predictions.

**Supplementary Table 4. CRISPR-Cas information of rMAGs.** Summary of CRISPR-Cas information per rMAG including number of CRISPR elements detected, sequence of CRISPR repeats and classification of the CRISPR-Cas system.

**Supplementary Table 5. Protospacer-containing contigs summary.** Number of spacer matching protospacers, before and after filtering procedures.

**Supplementary Table 5. Protospacer-containing contigs summary**

| Before search | | Unfiltered | *Filter 1 | **Filter 2 | Unique iMGE |
|---|---|---|---|---|---|
| **Unique Spacer** | 162,985 | 110,307 | 78,777 | 34,335 | - |
| **Non-unique (before redundancy removal)** | | | | | |
| **Protospacer** | NA | 18,599,466 | 2,400,670 | 750,375 | 209,199 |
| **PSCCs** | NA | 1,599,109 | 334,155 | 224,651 | 49,306 |

*** Filter 1: Filtering based on 95% identity and 95% query coverage**
**** Filter 2: Filtering sequences containing repeats**

**Supplementary Table 6**. **Summary of redundancy removal of iMGEs.** Number of candidate invasive mobile genetic elements (iMGEs) and protospacer-containing contigs (PSCC) before and after the removal of redundancy (using CD-HIT). The number of protospacers within the PSCCs is based on blastn matching of spacers and PSCC. Additionally, the classification of iMGEs as phage, plasmid, ambiguous and unclassified is based on predictions from VirSorter, VirFinder, PlasFlow and cBar.

**Supplementary Table 6. Summary of redundancy removal of iMGEs**

| **iMGE** | Redundant sequences | | Non-redundant sequences | | Proportion of redundancy (%) |
|---|---|---|---|---|---|
| | **With protospacer** | **Total** | **With protospacer** | **Total** | |
| Plasmids | 30183 | 1389477 | 18778 | 707093 | 45,187 |
| Phages | 3923 | 83733 | 2518 | 42039 | 48,974 |
| Ambiguous | 3662 | 46863 | 2401 | 80618 | 45,793 |
| Unclassified | 11538 | 11538 | 6663 | 6663 | 42,252 |
| Total | 49306 | 1531611 | 30360 | 836413 | 43,627 |

**Suplementary Table 7. Function within iMGEs.** COG categories identified in plasmids and phages. Number of genes and number of protospacers per functional category.

**Supplementary Table 8. Antibiotic resistant genes (ARGs) within iMGEs.** Summary of ARGs within iMGEs and iMGE-PSCCs.

**Supplementary Table 8. Antibiotic resistant genes (ARGs) within iMGEs.**

| | Plasmids | | Phages | |
|---|---|---|---|---|
| | **Number** | **Percentage** | **Number** | **Percentage** |
| Total | 707093 | 100 | 42039 | 100 |
| Number of MGEs containing at least one annotated gene | 516496 | 73,045 | 31283 | 74,414 |
| Number of MGE-PSCCs containing at least one annotated gene | 8977 | 1,270 | 1786 | 4,248 |
| Number of MGEs carrying at least one ARG | 1570 | 0,222 | 106 | 0,252 |
| Number of MGE-PSCCs carrying at least one ARG | 8 | 0,001 | 1 | 0,002 |
| Number of ARGs carried by MGEs | 1613 | 0,228 | 107 | 0,255 |
| Number of ARGs carried by unique MGEs | 38 | 0,005 | 9 | 0,021 |
| Number of ARGs carried by MGE-PSCCs | 8 | 0,001 | 1 | 0,002 |
| Number of ARGs carried by unique MGE-PSCCs | 5 | 0,001 | 1 | 0,002 |
| Number of ARGs containing at least protospacer | 3 | 0 | 0 | 0 |

**Supplementary Table 9. Specific ARGs within the iMGEs.** List of ARGs from ResFams database found within the iMGEs.

**Supplementary Table 10. Correlations between family-level groups**. Pearson correlations between MG-derived relative abundances of family-level bacterial, plasmid and phage groups, filtered by p-value ≤ 0.001. Columns correspond to the analysed time intervals, i.e. entire time-series, before, during, and after the community shift. The table shows correlations only if they are significant in selected time intervals, i) all the analyzed time intervals, ii) before, during and after the community shift, and not in the entire time-series, iii) before and during the community shift, and not in the other time intervals, iv) during and after the community shift, and not in the other time intervals, and v) before and after the community shift, and not in the other time intervals. Please refer to "Source Data Fig. 2a and Supplementary Fig. 4a" for all correlation values and their associated p-values.

**Supplementary Table 11. Summary of the linear models predicting *Microthrixaceae* bacterial abundance over time.** The analyzed time intervals, i.e. longer-term (entire time-series) as well as shorter-term intervals corresponding to before, during, and after the community shift. Statistical tests were two-sided.

**Supplementary Table 12. List of family-level bacteria, plasmid and phage groups within the optimal linear models**. The table contains the family-level groups that were significant within the linear models of *Microthrixaceae* bacterial family as response variable performed on the different time intervals, i.e. entire time-series as well as before, during, and after the community shift. Columns represent presence (1) or absence (0) per time interval, and the intersection of specific time intervals.

**Supplementary Table 13. Summary of spacers activity.** CRISPR spacer gain and loss events, summarized by type of targeted iMGE, i.e. plasmid or phage, and per microbial population.

**Supplementary Table 14: iMGE-host CRISPR based networks attributes.** This table contains the network properties of the plasmid-host and the phage-host CRISPR networks over time, i.e. network properties as number of nodes, number of interactions, modularity and nestedness over time, and node properties as betweenness, closeness and degree.

**Supplementary Table 15. One mode projection network from iMGE-CRISPR host networks.** The one-mode network represents the rMAGs, obtained from the plasmid- and phage-CRISPR host networks. The information within this table contains the number of common iMGEs between rMAGs and the network and node attributes.

**Supplementary Table 16. Summary information of spacers within rMAGs.** This table contains a summary of the final number of spacers within each rMAG containing CRISPRs, including spacers activity as gain and loss events.

## Supplementary Videos

**Supplementary Video 1**: Time-lapse plasmid-host network. Host nodes (circles) are coloured based on their taxonomy (See legend of Fig. 3).

**Supplementary Video 2**: Time-lapse phage-host network. Host nodes (circles) are coloured based on their taxonomy (See legend of Extended Data Fig. 7).

# Supplementary Notes

The supplementary notes contain detailed results, explanations and discussions.

## Supplementary Note 1: General assessment of the alignment of sequencing data

The MG and MT data provided the foundation for all the downstream analyses. We deemed it important to estimate the average community coverage based on the time-resolved MG and MT sequencing data. In this case, "community coverage" specifically refers to the estimated fraction of the genomes recovered in a given sequencing dataset, after accounting for factors such as community i) -richness, ii) -diversity and, most importantly, iii) sequencing depth[2]. The assessment was based on the output of Nonpareil[1,2] which estimates community coverage using unaligned (raw or pre-processed) sequencing reads. Specifically, we performed the assessment on IMP-based pre-processed MG and MT reads for each sample, rather than the raw sequencing reads, as the pre-processed reads are used for all downstream steps, including, but not limited to assembly, read mapping/alignment and inference of population sizes (rMAGs and iMGEs). Additionally, we performed the same assessment on the combined MG and MT data, given that IMP generates de novo assemblies based on these two data types[3]. On average, the combined MG and MT sequencing depth achieved approximately 50% community coverage (Supplementary Fig. 1 and Supplementary Table 1) which allowed detailed, population-level study of the prominent community members. In general, we observed that when MG and MT reads were combined, the coverage estimations typically fell somewhere in between the MG and MT coverage values. The detailed coverage values are available in Supplementary Table 1.

## Supplementary Note 2: Representative metagenome assembled genomes (rMAGs)

Given the time-resolved nature of our dataset, we aimed to link MAGs (i.e. bins) from different samples within the time-series that may be representative of the same microbial population, prior to downstream analyses. This was achieved through a systematic procedure to reduce the redundancy of the identified MAGs into representative MAGs (i.e. rMAGs), which are more suitable for downstream time-series analysis. The timepoint-specific binning was carried out using the same procedure described in Heintz-Buschart *et al.*[4]. Briefly, two-dimensional penta-nucleotide-based maps for all contigs $\geq$ 1kbp were generated using Vizbin[5] for assemblies of each time point. Maps from each time point were then clustered using the dbscan function in the R package fpc [6] using the same parameters described in Heintz-Buschart *et al.*[4]. Next, the number and multiplicity of 101 essential genes [7,8] were used to assess the completeness and contamination of clusters generated from dbscan. Clusters with multiple copies of the same essential genes were divided further by analysing the metagenomic coverage depth of the essential genes using the same settings utilized by Heintz-Buschart *et al.*[4]. The two last steps are repeated three times on overcomplete bins[4]. Accordingly, the quality of bins, based on their essential gene content are defined as follows: "P": more than 100/109 essential genes, less than 115 essential genes in total (<14% duplicated genes, >92% complete), "G": more than 71/109 essential genes (>65% complete), less than 20% in duplicate, "O": more than 51/109 essential genes (>47% complete), less than 20% in duplicate, "L": more than 31/109 essential genes (>28% complete), less than 20% in duplicate, "C": at least 1/109 essential genes (>= 1%

complete), less than 20% in duplicate, "E": no essential genes, "B": at least 1/109 essential genes (>= 1% complete), at least 20% in duplicate, "N": noise (https://git-r3lab.uni.lu/anna.buschart/MuStMultiomics/blob/master/autoCluster.R).

The binning procedure on all the timepoint assemblies yielded a total of 26,524 bins, hereafter. Based on the quality metrics established by Heintz-Buschart *et al.*[4], we selected 1,364 bins, now referred to as metagenomic species (MAGs) with quality criteria P, G, O and L and a collection of 85 isolate genomes for downstream dereplication[9]. The dereplication allowed us to link the selected MAGs and isolates from different time points. The dereplication process yielded 92 high-quality representative MAGs (i.e. rMAGs) which underwent taxonomic classification (detailed information of rMAGS available in Supplementary Table 3). Manual curation was carried out on rMAGs that were classified as *Candidatus* Microthrix parvicella. We then linked plasmids and phages to rMAGs based on the outcome of the binning cluster membership, i.e. if a plasmid or phage contig fell within a bin of a certain rMAG. Finally, we scanned the genomes for CRISPR operons, i.e. CRISPR loci with CRISPR-associated genes (*cas* genes)[10].

## Supplementary Note 3: Prediction of CRISPR elements

CRISPR information (i.e. repeats and spacers) were used to link host populations (i.e. rMAGs) to iMGEs. For this, we utilized two different tools to maximize the extraction of CRISPR information. First, CRASS[11] was used to extract CRISPR information (i.e. spacers, repeats and flanking sequences) directly from the preprocessed reads from IMP (both paired- and single-end reads). Next, we used metaCRT[12] to extract CRISPR information (i.e. spacers and repeats) on the contig level. We also used the contig-level information to extract flanking sequences from the metaCRT-derived CRISPR information to have equivalent information from both CRASS and metaCRT for further downstream processing.

Overall, CRASS predicted more spacers (Supplementary Fig. 2) while metaCRT predicted more repeats. This difference is likely due to the fact that spacers are more diverse elements, making direct extraction from sequencing reads particularly effective compared to extraction from the *de novo* assembled, consensus-based contigs. Nevertheless, the use of both tools yielded complementary, contextual information. On the one hand, extraction of CRISPR information from sequencing reads using CRASS was necessary because i) *de novo* assemblers do not resolve repetitive regions, such as CRISPRs effectively, and ii) such approaches allow resolution of CRISPR information from lowly abundant and/or rare populations. On the other hand, the extraction of repeats using metaCRT from contigs allows the resolution of the CRISPR loci and linking these to the constituent rMAGs. Overall, the combination of these methods allowed for the extraction of comprehensive information regarding the different CRISPR loci which, in turn, allowed for detailed assessment of spacer complements and their linking to targeted iMGEs.

We also inspected the representation of CRISPR elements on the MG and MT omic levels. In general, we found more repeats on the MT-level compared to the MG-level, while spacers were more abundant at the MG-level (Supplementary Fig. 2 and Extended Data Fig. 1). This may be due to the fact that the number of spacers is larger than the number of repeats. However, there may be different factors affecting the transcription of spacers including

differential abundance of sub-populations and/or differential expression between and/or within CRISPR arrays. In particular, leading spacers are typically more highly transcribed compared to their lagging counterparts.

A general assessment of the CRISPR elements highlighted that repeats had an average length of 30.9 bp (median=39 bp, SD=8.55 bp), while the shortest and longest sequences were 20 and 77 bp in length, respectively. In contrast, spacers had an average length of 33.22 bp (median=33, SD=6.26), whilst the shortest and longest spacers were 11 and 119 bps, respectively.

We proceeded to reduce the redundancy of the spacers using CD-HIT-EST and then BLASTN-searched (using parameters defined by previous work[13]) the unique set of spacers against all IMP-based MT-assembled and co-assembled contigs. The parts of contigs that matched to spacers were defined as protospacers, while the contigs were defined as protospacer containing contigs (PSCCs). Despite the removal of redundancy among the spacers and stringent criteria used within the previous BLASTN search, we further clustered the search results by 95% identity and 95% query coverage, followed by filtering contigs that contained repeats to ensure removal of any possible self-matches.

## Supplementary Note 4: Prediction of invasive mobile genetic elements (iMGEs)

We utilized an ensemble approach to predict putative iMGEs with specifically focusing on plasmids and bacteriophages (phages). The first approach relied on the CRISPR information in the form of spacer-protospacer complements (Supplementary Note 3). Additionally, we used VirSorter[14] and VirFinder[15] to predict sequences derived from phages. Finally, cBar[16] and PlasFlow[17] were used to predict plasmid-derived contigs.

We merged the results from all the aforementioned methods by assigning annotations to the sequences. A given sequence was annotated as a “plasmid” if it yielded a positive prediction by cBar or PlasFlow. Similarly, a sequence was annotated as “phage” if yielded a positive prediction by either VirSorter or VirFinder. A sequence was annotated as “ambiguous” if it was predicted as both plasmid and phage using any combination of the four aforementioned tools. However, all the aforementioned categories were not necessarily PSCCs because they had to contain at least one protospacer. Therefore, a contig was considered “unclassified” if it was a PSCC, but was not classified as a phage or a plasmid. Thereby, we extracted four classes of iMGEs annotated as either i) phage, ii) plasmid, iii) ambiguous or iv) unclassified. Supplementary Fig. 2 summarizes the outcome from the different methods and their classifications.

Interestingly, we found that sequences annotated as plasmids (707,093) outnumber phages (42,039) by around 16-fold. Additionally, 80,617 contigs were found to carry “ambiguous” predictions. 23,697 (2.86 %) of those annotated contigs (i.e. phage, plasmid, ambiguous) contained at least one protospacer. Furthermore, a small number of contigs with protospacers (6,663) were annotated as “unclassified” due to their lack of plasmid and/or phage prediction. Overall, the total number of annotated sequences comprised 6.97 % of the IMP-based co-assembled contigs.

The redundancy of the identified iMGE sequences were reduced by clustering all the annotated sequences using CD-HIT-EST[18]. Upon clustering, the original annotations of the cluster representatives were retained. The non-redundant set of iMGEs retained similar

proportions to those in the redundant set, i.e. approximately 17-fold more plasmids than phages. Importantly, a total of 30,360 unique PSCCs were retained for further analysis of iMGEs and the associated host dynamics. Supplementary Table 6 summarizes the redundant and unique (non-redundant) set of iMGEs that were predicted from the analyses. Finally, the absence of prophage predictions could be explained by the fact that i) VirSorter was the only tool that we applied which was capable of predicting prophage sequences[14] , ii) short assembly contigs, and iii) limited prophage sequences within public databases for this specific environment.

## Supplementary Note 5: Functional analysis of PSCCs

We inspected if CRISPR systems/immunity targeted genes with specific functions[19,20] within iMGEs. For this, we predicted annotated gene functions within both non-redundant plasmids and phages based on KEGG identifiers and linked those identifiers to the corresponding COG categories. The most frequently (top 4) targeted functional categories within plasmids containing protospacers (plasmid-PSCCs) were "Replication, recombination and repair", "General function prediction only", "Transcription" and "Mobilome: prophages, transposons" (Extended Data Fig. 2). The latter highlighting the potential limitations of phage prediction methods. However, the category of genes most enriched in protospacers was "Nucleotide transport and metabolism", which was not present within the top 4 functional categories of plasmid-PSCCs (Supplementary Table 7). Specific gene functions within this category include "Adenosine deaminase". The most frequently targeted phages (phage-linked PSCCs) also contained the same top 4 categories as plasmid-PSCCs, but differed in their order (Extended Data Fig. 2). Unlike plasmids-PSCCs, the category of genes containing the highest number of protospacers was "Replication, recombination and repair" and "Mobilome: prophages, transposons", which were the two most frequently targeted categories within phage-PSCCs. Gene functions within these categories included "DNA modification methylase" and "Phage terminase large subunit", respectively. Generally, protospacers were overrepresented within genes that play essential roles in replication, retention and transmission of the corresponding iMGEs. We also inspected the lists for potential depletions in functional categories. In general, we observed that the most depleted (i.e. <1% in PSCCs compared to non-PSCCs) categories were "RNA processing and modification", "Extracellular structures", "Secondary metabolites biosynthesis, transport and catabolism" and "Antimicrobial resistance: ResFam". Detailed information of the specific gene functions based on their COG categories can be found via Zenodo[21]. Using this broader functional analysis, we did not identify categories depleted specifically when comparing phages to plasmids. Overall, the proportion of targeted genes within the PSCC of phages is higher (30%) compared to their plasmid counterparts (25%), likely due to the lower cargo carrying capacity and dense coding regions of the phage genomes when compared to plasmids[22].

## Supplementary Note 6: Correlation analysis

In general, temporal dynamics were analyzed based on the entire time-series, i.e. 2011-03-21 to 2012-05-03. However, we also inspected the shorter-term temporal dynamics by manually defining three overlapping shorter-term intervals. These intervals are based on the shift in community structure when the abundance of *Microthrixaceae* family decreases drastically as a reference point. The intervals also overlapped to ensure that sufficient data points were available for the downstream analyses of localised temporal dynamics. Accordingly, the intervals were defined as: i) before shift: 2011-03-21 to 2011-08-29, ii) during shift: 2011-08-05 to 2011-01-19, and after shift: 2011-12-21 to 2012-05-03 (Fig. 2).

MAGs, plasmids and phages were merged on the family-level (i.e. family-level groups). Plasmids and phages that could not be assigned to any family-level group were collapsed into their own distinct groups (i.e., "plasmid_NA" and "phage_NA"). We calculated the Pearson correlation between the defined bacterial, plasmid and phage groups (all versus all). Next, a hierarchical clustering on the Euclidean distances was applied. This resulted in a total of six clusters. Extended Data Fig. 4 and Supplementary Figure 5 show the cluster memberships and dynamics of the groups.

Based on the correlations, we first observed the cluster-level dynamics of the entire time-series). The dominant clusters 2, 3 and 4 were markedly affected by the community shift in the period between 2011-10-05 and 2012-01-11, during which the abundance of cluster 3 increased significantly, while clusters 2 and 4 reduced significantly, corresponding to the drastic reduction in the abundance of *Microthrixaceae*. Cluster 5 exhibited a peak on 2011-08-19, followed by a gradual decrease until the aforementioned community shift (i.e. drop in *Microthrixaceae*). Interestingly, cluster 4 peaked on 2011-09-28, while cluster 2 peaked just prior to the community shift on 2011-10-12 (Extended Data Fig. 4 and Supplementary Fig. 5).

## Supplementary Note 7: Linear models

To further investigate the dynamics, we developed linear models based on the dominant *Microthrixaceae* as the representative family (i.e., the response variable), including a random sampling approach for linear model identification (see Material and Methods). Similar to the correlation above, linear models were applied based on the entire time-series and the predifined shorter-term intervals (Fig.2, Extended Data fig. 6, Supplementary Fig. 6, Supplementary Table 11). We assessed the quality of the models with the distribution of the adjusted $R^2$ values. We observed a bimodal shape with a high number of optimal models and a long tail for non-predictive models (Extended Data fig. 5). Next, we selected the models with the highest adjusted $R^2$ values, and inspected these for potential enrichments in specific family-level groups, to select the best models. We observed that plasmids of *Microthrixaceae* were present in 100% of the best models (Extended Data Fig. 5). We subsequently analyzed a globally optimal model with an adjusted $R^2$ value of 0.9983. In agreement with the enrichment analysis, the plasmids of *Microthrixaceae* and iMGEs assigned at family level, such as *Saprospiraceae* and *Moraxellaceae*, exhibited significant contributions, while *Cryomorphaceae* plasmids and phages did not exhibit any significant contribution. We subsequently excluded non-significant families from the best global model, which led to a reduced model with an adjusted $R^2$ value of 0.997 and, compared to the global model, did not exhibit a significant decrease in the

adjusted $R^2$ value (Supplementary Table 11). Overall, the longitudinal abundance data for *Microthrixaceae* exhibits good agreement in those models (Fig. 2).

To further validate the observed patterns, we repeated the linear modelling of the shorter-term intervals using the same procedure as for the models of the entire time-series. We obtained $R^2$ values of 1 in all the global short-term models, with no significant reductions in the $R^2$ values for the reduced models (Supplementary Table 11). We found that the plasmids of *Microthrixaceae* appeared as the only common significant predictor in all the models (entire time-series and short-term intervals). To further assess the relative importance of this group as main predictor of the *Microthrixaceae* family abundance dynamics, we specifically investigated the dependence of the model qualities with respect to *Microthrixaceae* plasmids (Supplementary Table 12). For this purpose, we excluded *Microthrixaceae* plasmids from the respective reduced models, which led to a reduction in predictive power in all models, especially in the short-term model before (from $R^2$=0.99 to $R^2$=0.21) the community shift. In contrast to the dynamics before and during the community shift, we found that the shorter-term models highlighted *Microthrixaceae* and *Moraxellaceae* phages as significant predictors after the community shift.

Overall, the actual longitudinal abundance data for *Microthrixaceae* is in good agreement with the global and reduced models, but less so when removing *Microthrixaceae* plasmids as predictors (Fig. 2, Extended Data Fig. 6 and Supplementary Fig. 6). Thereby, plasmids exhibited stronger effect on the prediction of *Microthrixaceae* abundances compared to phages. This in turn indicates a higher relative importance for plasmids in governing the *Microthrixaceae* dynamics.

## Supplementary Note 8: iMGE-CRISPR-host based networks

The constructed iMGE-host networks are bipartite networks, i.e. there are two groups of nodes (rMAGs and iMGEs), where the considered interactions occur between elements of the different groups and not between elements of the same group (rMAG-iMGEs). Properties of bipartite networks include modularity and nestedness. Modularity (Q) relates to the connectivity between different groups[23], i.e. in this study it reflects connectivity between groups of iMGEs (phage or plasmid) and groups of hosts. On the other hand, nestedness is given as the value of the "Nestedness matrix based on Overlap and Decreasing Fill" (NODF) and represents the measure of structure in an ecological system, i.e. in this study it reflects the iMGE host range specificity[24].

The global phage-host network had a modularity Q of 0.7 (out of 1) and a nestedness NODF of 1.0 (out of 100), with similar values observed in the time point-specific networks (Supplementary Table 14). Similarly, the global plasmid-host network had a modularity and nestedness of Q=0.77 and NODF=3.43, respectively (Supplementary Table 14). These network properties indicate high compartmentalization, i.e. specific groups of iMGEs interact with specific hosts, and a restricted host range. However and interestingly, the host range of plasmids is broader than for phages (Supplementary Fig. 8).

Finally, it is important to note that these properties were exclusive for iMGE-host interactions via CRISPR, compared to networks including all interacting MGEs, not limited to those interacting via CRISPRs but also using other mechanisms.

## Supplementary Note 9: The CRISPR-Cas genes of *Candidatus* Microthrix parvicella Bio-17

A complete *Candidatus* Microthrix parvicella CRISPR operon was also detected within a single contig of 10,224 bp (D47_L1.43.1_contig_476300). Specifically, the CRISPR operon contained i) CRISPR-associated endonuclease Cas1, ii) CRISPR-associated endonuclease Cas2, iii) CRISPR-associated endonuclease/helicase Cas3, iv) CRISPR-associated protein Cas7, v) CRISPR-associated proteins Cas8, vi) *csb2gr5* and vii) a CRISPR locus with 11 repeats similar to those encoded by the *M. parvicella* Bio17-1 genome[25]. This combination of *cas* genes defined the CRISPR-Cas system as a type I and subtype I-U, whose signature genes are *cas3HD* and *cas8u*, respectively[26–28]. Cas1 and Cas2 are universal proteins involved in CRISPR-spacer insertion[26]. Furthermore, Cas7, Cas3HD and Cas8u1 are all involved in the interference step of CRISPR-based immunity, while the function of Csb2gr5 remains unclear[26,28]. To ensure accuracy of the predicted CRISPR operon, we processed the genomes of *Candidatus* Microthrix parvicella Bio-17 and the contig containing the CRISPR operon with CRISPRCasFinder[29], where we further confirmed a highly similar CRISPR operon[30]. The *cas* genes were found to be expressed at both the MT- and MP-levels while we observed spacer gain and loss events within the CRISPR locus during the time-series, which points towards an active CRISPR system (Figs. 4 and 5). Specifically, we detected 31 spacer gain events for which iMGE sequences were detected before their linked spacers, 9 spacer gain events for which iMGE sequences were detected at the same time as the spacers, and 5 spacer gain events for which the iMGE sequences were detected after their linked spacers. Finally, the average lag time of an integration event (i.e. time between detection of iMGE and detection of spacer) was 6 weeks (median=1, SD=12) for spacers targeting plasmid sequences and 4 weeks (median=1, SD=7) for spacers targeting phage sequences.

## Supplementary Note 10: Contrasting *Candidatus* Micorthrix parvicella's spacers and iMGEs with other populations

The *M. parvicella*-like rMAG-165 (hereafter referred to as *M. parvicella*) clearly demonstrated activity of its CRISPR system in terms of gene and protein expression, as well as spacer gain and loss activity (Supplementary Note 9). However, we were unable to assess the magnitude of the CRISPR system's activity in terms of iMGE targeting. Therefore, we performed an additional assessment to contrast CRISPR system activity of *M. parvicella* with other populations. Accordingly, we sourced additional rMAGs that encoded complete CRISPR systems by fulfilling the following criteria: i) CRISPR locus, ii) a set of *cas* genes, iii) CRISPR system type prediction and iv) occurrence within a single contig. Subsequently, we found rMAG-31 and rMAG-40, classified as *Intrasporangium calvum* and *Leptospira biflexi*, respectively, as suitable candidates for this assessment.

rMAG-31 (*I. calvum*) encoded a type I CRISPR operon which in turn encoded seven *cas* genes and one CRISPR locus encoded on a single contig of 24,304 bp. However, the *cas* genes and Cas proteins were found to be lowly expressed, relative to those of *M. parvicella*. Its CRISPR locus contained 129 spacers, with only 7 spacers targeting iMGE sequences within the time-series, exclusively plasmid sequences. Spacer integration events were lower when

compared to *M. parvicella* with only one spacer gain event occurring in 18 weeks (Supplementary Tables 13 and 16).

rMAG-40 (*L. biflexi*) encoded a type V CRISPR operon which contained four *cas* genes (Extended Data Fig. 10) and one CRISPR locus which was encoded on a single contig of 12,586 bp. The expression of *cas* genes was found to be comparable to *M. parvicella*, but the expression of Cas proteins was lower. Conversely, spacer integration events were significantly more frequent compared to *M. parvicella*, with rMAG-40 exhibiting acquisition of both plasmid- and phage-derived spacers alike. Plasmid-based spacer integration was the most prevalent (Supplementary Table 16). The average time for the integration of spacers targeting plasmids was 12 weeks (median=6, SD=15), while for spacers targeting phages was 11 weeks (median=5, SD=14) (Supplementary Table 13).

In summary, we compared the CRISPR system activity of *M. parvicella* with other rMAGs, and observed that rMAG-31 (*I. calvum*) had lower activity compared *M. parvicella*. On the other hand, rMAG-40 demonstrated lower levels of CRISPR system activity when compared to *M. parvicella*, in terms of gene and protein expression, despite demonstrating enhanced spacer integration dynamics, both in terms of frequency (i.e. absolute number) and breadth (i.e. types of potential iMGEs targeted). In general, we show that different population-level CRISPR-Cas dynamics exist at the level of gene and protein expression as well as spacer integration activity. Based on our results, *M. parvicella* populations do contain a functional CRISPR system, but uses it rather sparingly compared to other population such as rMAG-40 (*L. biflexi*).

# Bibliography


1. Rodriguez-R, L. M. & Konstantinidis, K. T. Nonpareil: a redundancy-based approach to assess the level of coverage in metagenomic datasets. *Bioinformatics* **30**, 629–635 (2014).
2. Rodriguez-R, L. M. & Konstantinidis, K. T. Estimating coverage in metagenomic data sets and why it matters. *ISME J.* **8**, 1–3 (2014).
3. Narayanasamy, S. *et al.* IMP: a pipeline for reproducible reference-independent integrated metagenomic and metatranscriptomic analyses. *Genome Biol.* **17**, 260 (2016).
4. Heintz-Buschart, A. *et al.* Integrated multi-omics of the human gut microbiome in a case study of familial type 1 diabetes. *Nat. Microbiol.* **2**, 16180 (2016).
5. Laczny, C. C. *et al.* VizBin - an application for reference-independent visualization and human-augmented binning of metagenomic data. *Microbiome* **3**, 1 (2015).
6. Procedures, T. F. *et al.* Package ‘ fpc ’. (2019).
7. Dupont, C. L. *et al.* Genomic insights to SAR86, an abundant and uncultivated marine bacterial lineage. *ISME J* **6**, (2012).
8. Albertsen, M. *et al.* Genome sequences of rare, uncultured bacteria obtained by differential coverage binning of multiple metagenomes. *Nat Biotechnol* **31**, (2013).
9. Olm, M. R., Brown, C. T., Brooks, B. & Banfield, J. F. dRep: a tool for fast and accurate genomic comparisons that enables improved genome recovery from metagenomes through de-replication. *ISME J.* **11**, 2864–2868 (2017).
10. Zhang, Q. & Ye, Y. Not all predicted CRISPR–Cas systems are equal: isolated cas genes and classes of CRISPR like elements. *BMC Bioinformatics* **18**, 92 (2017).
11. Skennerton, C. T., Imelfort, M. & Tyson, G. W. Crass: Identification and reconstruction of CRISPR from unassembled metagenomic data. *Nucleic Acids Res.* **41**, (2013).
12. Bland, C. *et al.* CRISPR recognition tool (CRT): a tool for automatic detection of clustered regularly interspaced palindromic repeats. *BMC Bioinformatics* **8**, 209 (2007).
13. Biswas, A., Gagnon, J. N., Brouns, S. J. J., Fineran, P. C. & Brown, C. M. CRISPRTarget. *RNA Biol.* **10**, 817–827 (2013).
14. Roux, S., Enault, F., Hurwitz, B. L. & Sullivan, M. B. VirSorter: mining viral signal from microbial genomic data. *PeerJ* **3**, e985 (2015).
15. Ren, J., Ahlgren, N. A., Lu, Y. Y., Fuhrman, J. A. & Sun, F. VirFinder: a novel k-mer based tool for identifying viral sequences from assembled metagenomic data. *Microbiome* **5**, 69 (2017).
16. Zhou, F. & Xu, Y. cBar: a computer program to distinguish plasmid-derived from chromosome-derived sequence fragments in metagenomics data. *Bioinformatics* **26**, 2051–2052 (2010).
17. Krawczyk, P. S., Lipinski, L. & Dziembowski, A. PlasFlow: predicting plasmid sequences in metagenomic data using genome signatures. *Nucleic Acids Res.* **46**, e35 (2018).
18. Fu, L., Niu, B., Zhu, Z., Wu, S. & Li, W. CD-HIT: accelerated for clustering the next-generation sequencing data. *Bioinformatics* **28**, 3150–3152 (2012).
19. Shmakov, S. A. *et al.* The CRISPR Spacer Space Is Dominated by Sequences from Species-Specific Mobilomes. *MBio* **8**, e01397-17 (2017).
20. Davison, M., Treangen, T. J., Koren, S., Pop, M. & Bhaya, D. Diversity in a Polymicrobial Community Revealed by Analysis of Viromes, Endolysins and CRISPR Spacers. *PLoS One* **11**, e0160574 (2016).
21. Tatusov, R. L., Galperin, M. Y., Natale, D. A. & Koonin, E. V. The COG database: a

tool for genome-scale analysis of protein functions and evolution. *Nucleic Acids Res.* **28**, 33–6 (2000).
22. Leclercq, S., Gilbert, C. & Cordaux, R. Cargo capacity of phages and plasmids and other factors influencing horizontal transfers of prokaryote transposable elements. *Mob. Genet. Elements* **2**, 115–118 (2012).
23. Newman, M. E. J. Modularity and community structure in networks. *Commun. Law* **19**, 56–62 (2006).
24. Koskella, B. & Meaden, S. Understanding bacteriophage specificity in natural microbial communities. *Viruses* **5**, 806–823 (2013).
25. Muller, E. E. L. *et al.* Genome sequence of 'Candidatus Microthrix parvicella' Bio17-1, a long-chain-fatty-acid-accumulating filamentous actinobacterium from a biological wastewater treatment plant. *J. Bacteriol.* **194**, 6670–6671 (2012).
26. Makarova, K. S. & Koonin, E. V. Annotation and Classification of CRISPR-Cas Systems. *Methods Mol. Biol.* **1311**, 47–75 (2015).
27. Crawley, A. B., Henriksen, J. R. & Barrangou, R. CRISPRdisco: An Automated Pipeline for the Discovery and Analysis of CRISPR-Cas Systems. *Cris. J.* **1**, 171–181 (2018).
28. Koonin, E. V, Makarova, K. S. & Zhang, F. Diversity, classification and evolution of CRISPR-Cas systems. *Curr. Opin. Microbiol.* **37**, 67–78 (2017).
29. Couvin, D. *et al.* CRISPRCasFinder, an update of CRISRFinder, includes a portable version, enhanced performance and integrates search for Cas proteins. *Nucleic Acids Res.* **2**, W246–W251 (2018).
30. Martinez Arbas, S. & Narayanasamy, S. CRISPR locus information of M. parvicella in Martinez Arbas, Narayanasamy et. al. (2020). (2020). doi:10.5281/ZENODO.3766442
