# Supplementary material for: Roles of bacteriophages, plasmids and CRISPR immunity in microbial community dynamics revealed using time-series integrated meta-omics
Source: Nat Microbiol. 2020 Nov 2;6(1):123–35. doi: 10.1038/s41564-020-00794-8 (PMC7752763; doi:10.1038/s41564-020-00794-8)
Supplement: Supplementary file 2 — Reporting Summary [file 41564_2020_794_MOESM2_ESM.pdf]

nature research

Corresponding author(s): Paul Wilmes

Last updated by author(s): 18/08/2020

Reporting Summary

Nature Research wishes to improve the reproducibility of the work that we publish. This form provides structure for consistency and transparency in reporting. For further information on Nature Research policies, see Authors & Referees and the Editorial Policy Checklist.

Statistics

For all statistical analyses, confirm that the following items are present in the figure legend, table legend, main text, or Methods section.

n/a	Confirmed	
☐	☒	The exact sample size (n) for each experimental group/condition, given as a discrete number and unit of measurement
☐	☒	A statement on whether measurements were taken from distinct samples or whether the same sample was measured repeatedly
☐	☒	The statistical test(s) used AND whether they are one- or two-sided<br>*Only common tests should be described solely by name; describe more complex techniques in the Methods section.*
☐	☒	A description of all covariates tested
☐	☒	A description of any assumptions or corrections, such as tests of normality and adjustment for multiple comparisons
☐	☒	A full description of the statistical parameters including central tendency (e.g. means) or other basic estimates (e.g. regression coefficient) AND variation (e.g. standard deviation) or associated estimates of uncertainty (e.g. confidence intervals)
☐	☒	For null hypothesis testing, the test statistic (e.g. F, t, r) with confidence intervals, effect sizes, degrees of freedom and P value noted<br>*Give P values as exact values whenever suitable.*
☒	☐	For Bayesian analysis, information on the choice of priors and Markov chain Monte Carlo settings
☒	☐	For hierarchical and complex designs, identification of the appropriate level for tests and full reporting of outcomes
☐	☒	Estimates of effect sizes (e.g. Cohen's d, Pearson's r), indicating how they were calculated

Our web collection on statistics for biologists contains articles on many of the points above.

Software and code

Policy information about availability of computer code

Data collection	Base calling of sequenced metagenomic (MG) and metatranscriptomic (MT) was processed using commercial software bundled within Illumina sequencing platforms to generate raw FASTQ data. Raw metaproteomic (MP) mass spectra were acquired using commercial software from Thermo Fischer Scientific.<br><br>This work represents part of a larger ongoing multi-annual project. Please refer previous publications for detailed information on NGS and mass spectrometry platforms and the associated software for those platforms:<br>https://doi.org/10.1038/ismej.2012.72<br>https://doi.org/10.1038/ncomms6603<br>https://doi.org/10.1038/npjbiofilms.2015.7<br>https://doi.org/10.1186/s40793-017-0274-y
Data analysis	All the code related to this work is available in three separate repositories:<br>i) Integrated Meta-omics Pipeline (IMP), binning and population genomes: https://git-r3lab.uni.lu/shaman.narayanasamy/LAO-time-series,<br>ii) CRISPR and mobile genetic element analyses: https://git-r3lab.uni.lu/susana.martinez/LAO_multiomics_CRISPR_iMGEs,<br>iii) for the isolate assembly analyses: https://git-r3lab.uni.lu/shaman.narayanasamy/Isolate_analysis/activity.<br>This information is included in the manuscript in the "Code availability" section.<br><br>The software (and versions) used within this work include:<br>IMP (ver. 1.3)<br>Nonpareil (ver 2.0)<br>Graph2Pro (no ver. number available)<br>dRep (ver. 0.5.4)<br>CheckM (ver. 1.0.7)<br>R statistical package (ver. 3.4.1)

Cytoscape (ver 3.6.1)
bwa (ver. 0.7.17)
Crass (ver. 0.3.8)
metaCRT (no ver. number available)
CD-HIT (ver. 4.6.7)
VirSorter (ver. 1.0.3)
VirFinder (ver 1.0.0)
PlasFlow (ver 1.0.7)
cBar (1.2)
snakemake (ver from 3.10.2 to 5.1.4)

For manuscripts utilizing custom algorithms or software that are central to the research but not yet described in published literature, software must be made available to editors/reviewers. We strongly encourage code deposition in a community repository (e.g. GitHub). See the Nature Research guidelines for submitting code & software for further information.

Data

Policy information about availability of data

All manuscripts must include a data availability statement. This statement should provide the following information, where applicable:

- Accession codes, unique identifiers, or web links for publicly available datasets
- A list of figures that have associated raw data
- A description of any restrictions on data availability

The metagenomic and metatranscriptomics FASTQ files, rMAGs, and isolate genomes are available as NCBI BioProject PRJNA230567 (https://www.ncbi.nlm.nih.gov/bioproject/?term=PRJNA230567). MP data has been deposited in the PRIDE database under the accession number PXD013655 (https://www.ebi.ac.uk/pride/archive/projects/PXD013655). Supplementary Files 1 (https://doi.org/10.5281/zenodo.3774024) and 2 (https://doi.org/10.5281/zenodo.3766442) are available via Zenodo.
Additional publicly available projects cited by this work include NCBI BioProject PRJNA174686 (https://www.ncbi.nlm.nih.gov/bioproject/?term=PRJNA174686).

Field-specific reporting

Please select the one below that is the best fit for your research. If you are not sure, read the appropriate sections before making your selection.

☐ Life sciences ☐ Behavioural & social sciences ☒ Ecological, evolutionary & environmental sciences

For a reference copy of the document with all sections, see nature.com/documents/nr-reporting-summary-flat.pdf

Ecological, evolutionary & environmental sciences study design

All studies must disclose on these points even when the disclosure is negative.

Study description	A generation-resolved, integrated meta-omic analysis of invasive mobile genetic elements and microbial host dynamics within a microbial community from a biological wastewater treatment plant spanning one and a half years.
Research sample	Individual floating sludge islets from the surface of the anoxic tank of the Schifflange biological wastewater treatment plant were sampled due to their richness in lipid accumulating organisms. They were then subjected to a concomitant biomolecular extraction of DNA, RNA an proteins, and a high throughput measurements to obtain metagenomic, metatranscriptomic and metaproteomic datasets to be computationally analysed.
Sampling strategy	Samples were collected from Schifflange biological wastewater treatment plant (Esch-sur-Alzette, Luxembourg; 49°30′48.29″N; 6°1′4.53″E). Individual floating sludge islets were collected from the same spot of the anoxic tank, along with physico-chemical parameters of the water, i.e. pH, temperature, conductivity, oxygen.<br>Two initial samples were collected on 2010-10-04 and 2011-01-25 in the context of previously published work (https://doi.org/10.1038/ncomms6603 and https://doi.org/10.1038/npjbiofilms.2015.7). More frequent sampling was performed from 2011-03-21 to 2012-05-03, of which data from three samples (2011-10-05, 2011-10-05 and 2012-01-11) have been previously published (https://doi.org/10.1038/ncomms6603). A total of 53 samples were collected over a period of 578 days. The mean sample frequency was 8 days (SD=16 days). The sampling procedure was designed to span at least one entire annual seasonal cycle (i.e. winter, spring, summer, autumn) while the sampling frequency corresponded to the doubling time of the dominant bacterial population of approximately 8 days, thus representing an approximate generational time scale.<br>Sampling was performed by Laura A. Lebrun and Emilie E.L. Muller.<br>This work represents part of a larger ongoing multi-annual project. Thus, all the samples were subjected to the same experimental protocols. Please refer to detailed methods on sampling procedures in previous publications:<br>https://doi.org/10.1038/ncomms6603<br>https://doi.org/10.1038/npjbiofilms.2015.7
Data collection	Laura A. Lebrun and Emilie E.L. Muller performed the concomitant biomolecular extractions resulting in fractions of DNA, RNA, proteins and metabolites for each in situ sample. They also performed the bacterial strain isolation (re-plating), screening and genomic DNA extraction for lipid accumulating bacteria.<br>Nathan D. Hicks, Cindy M. Liu, Lance B. Price, John D. Gillece, James M. Schupp and Paul S. Keim performed the DNA and RNA library preparation and next-generation sequencing (NGS) to obtain MG and MT data. They also performed the DNA library preparation and NGS of isolate genomic data.

Michael R. Hoopmann and Robert L. Moritz performed the mass-spectrometry measurements of the protein fractions.
This work represents part of a larger ongoing multi-annual project. For detailed information and descriptions about data collection, experimental protocols, experimental kit versions, DNA and RNA library preparation, proteomic sample preparation, high-throughput platforms, please refer to the following articles:
https://doi.org/10.1038/ismej.2012.72
https://doi.org/10.1038/ncomms6603
https://doi.org/10.1038/npjbiofilms.2015.7
https://doi.org/10.1186/s40793-017-0274-y

Timing and spatial scale

Individual floating sludge islets within anoxic tank number one of the Schifflange BWWT plant (Esch-sur-Alzette, Luxembourg; 49° 30'48.29"N; 6°1'4.53"E) were sampled always on the same spot. Sampling was carried out from 2010-10-04 to 2012-05-03. Two samples were collected on 2010-10-04 and 2011-01-25, to determine the sequencing conditions and the microbial diversity and was published in previous work. Subsequently, samples were collected on a weekly basis from 2011-03-21 to 2012-05-03, which approximately corresponds to the generational time scale of the sludge of eight days. The lack of samples in periods; from 2011-07-08 to 2011-08-05, from 2011-10-12 to 2011-11-02, and from 2011-11-20 to 2012-12-21 are due to absence of foaming islets as consequence of (i) heavy or continued rain and/or (ii) natural decrease of foam during summer and autumn seasons.

Data exclusions

The first two samples, collected on 2010-10-04 and 2011-01-25, were excluded from the all analyses after the "population abundance estimation" (in the "Binning, selection of representative genomic bins, taxonomy and estimation of abundance" section) because the sampling occurred before the period of weekly sample collection (i.e. 2011-03-21 to 2012-05-03) and therefore did not fit within the generational time-scale.

Reproducibility

Experimental procedures adhered to previously published protocols. Open source software was used in all the computational analyses. All custom scripts and commands are available within multiple Gitlab repositories. Wherever applicable, the software versions are reported in "Methods and Material" within the manuscript.

Randomization

Samples collected from 2011-03-21 to 2012-05-03 were randomized before biomolecular extractions. The biomolecular fractions were further randomized prior to the high-throughput measurements.
The two initial samples, collected on 2010-10-04 and 2011-01-25, were not included within the aforementioned randomization procedure(s) as they were collected in the context of previous work (https://doi.org/10.1038/ncomms6603 and https://doi.org/10.1038/npjbiofilms.2015.7) and were used to pilot the experimental protocols which was conducted prior to the higher frequency sampling (i.e. from 2011-03-21 to 2012-05-03).

Blinding

Blinding is not applicable in this study as it did not involve human subjects, but rather data from in situ samples from a naturally occurring environment.

Did the study involve field work? ☒ Yes ☐ No

Field work, collection and transport

Field conditions

Anoxic tank of an activated sludge (biological) wastewater treatment facility under seasonal climatic conditions (i.e. spring, summer, autumn and winter).

Location

Schifflange biological wastewater treatment plant (Esch-sur-Alzette, Luxembourg; 49°30′48.29″N; 6°1′4.53″E).

Access and import/export

Access was granted to the research personnel based on agreement between the principal investigator, Prof. Paul Wilmes (on behalf of the research institution), and the wastewater treatment facility management (Mr. Bissen and Mr. Di Pentima) from the Syndicat Intercommunal a Vocation Ecologique (SIVEC), Schifflange, Luxembourg. All research personnel are informally introduced to the management and personnel of the facility prior to conducting any work. Research personnel were not provided with keys or electronic access cards, and thus could only enter the premises upon the permission of personnel at the entrance of the facility.

Disturbance

Sampling had a minimum-to-no impact on the operations of the wastewater treatment facility. The work of the researchers did not require (complete or partial) shutdown or any operational disruption of the facility. Sampling was performed by the research personnel (Emilie E.L. Muller and Laura A. Lebrun) without any involvement of the staff of the facility. Research personnel either brought their own equipment or used equipment from the site, which was dedicated to them, thus not hindering any operations or personnel within facility. Researchers could access operational readings (e.g. temperature, inflow, outflow, etc.) of the facility directly via a dedicated web portal of the facility using login credentials provided by the facility management. Two formal meetings weres organized between researchers and management of the facility over the past five years.

Reporting for specific materials, systems and methods

We require information from authors about some types of materials, experimental systems and methods used in many studies. Here, indicate whether each material, system or method listed is relevant to your study. If you are not sure if a list item applies to your research, read the appropriate section before selecting a response.

Materials & experimental systems

n/a	Involved in the study
☒	☐ Antibodies
☒	☐ Eukaryotic cell lines
☒	☐ Palaeontology
☒	☐ Animals and other organisms
☒	☐ Human research participants
☒	☐ Clinical data

Methods

n/a	Involved in the study
☒	☐ ChIP-seq
☒	☐ Flow cytometry
☒	☐ MRI-based neuroimaging
